# Fire risk assessment for high-rise buildings based on Integrated Disaster Risk Theory

**DOI:** 10.1371/journal.pone.0358941

**Published:** 2026-10-05

**Authors:** Yingda Huang, Zonglai Liu, Xiaogeng Wei, Zhi-Jin Wu

**Affiliations:** 1 College of Jilin Emergency Management, Changchun Institute of Technology, Changchun, China; 2 China City Development Academy, Beijing, China; 3 Jilin Heyuan Fire Engineering Co., Ltd., Changchun, China; Beijing Technology and Business University, CHINA

## Abstract

High-rise buildings have become a dominant form of urban development. However, because these buildings face multiple fire safety risks, including difficulties in fire suppression, diverse occupancy types, rapid fire spread, and challenging evacuation, once a fire occurs, it is likely to cause serious casualties and significant property losses. It is therefore essential to carry out fire risk assessments for high-rise buildings in advance and to adopt targeted prevention and control measures. To improve the systematic and scenario-specific assessment of fire risk in high-rise buildings, this study introduces the Integrated Disaster Risk Theory into high-rise building fire risk assessment and reconstructs the assessment indicator system according to the specific formation mechanism of high-rise building fires. Indicator weights were integrated using a game-theoretic approach and then combined with cloud model theory to construct a fire risk assessment model for high-rise buildings, thereby identifying the corresponding fire risk levels of high-rise buildings. The assessment results for a typical high-rise building in Changchun showed that its fire risk level was Grade II, indicating a relatively low fire risk. This result was consistent with those obtained using an existing fire risk index method (FRI), thereby supporting the plausibility and consistency of the proposed model. This study provides a more targeted fire risk assessment framework for high-rise buildings, offering methodological support for fire prevention, evacuation management, and emergency rescue resource allocation.

## 1. Introduction

With the growing siphon effect of cities on surrounding populations, urban population density continues to rise. To meet residents’ daily needs and improve land-use efficiency, the number of high-rise buildings has increased sharply. While improving urban functionality, this development has also introduced new fire safety challenges.

The definition of a high-rise building differs across countries. In the United States, buildings with a height of 24.6 m or more, or with seven or more floors, are generally classified as high-rise; in Japan, the corresponding threshold is 31 m or eight or more floors; in the United Kingdom, buildings with a height equal to or greater than 24.3 m are regarded as high-rise. In China, high-rise buildings are defined as residential buildings taller than 27 m, as well as non-single-storey industrial buildings, warehouses, and other civil buildings taller than 24 m [1]. Because such buildings typically have a large overall volume, complex structural layouts, high occupant density, substantial fire load, and large quantities of combustible materials, they present significant fire safety hazards: once a fire starts, it can spread rapidly, become difficult to bring under control, and lead to major economic losses and serious casualties [2]. Between 2008 and 2023, 115,993 fires were reported in high-rise buildings in China, an average of 7,250 incidents per year, with a clear upward trend over time. In 2023 alone, 23,633 high-rise building fires occurred, and in the first nine months of 2024 the number had already reached 38,750 [3,4]. These data point to the need to develop a fire risk assessment system for high-rise buildings that can assign appropriate risk levels, a task of considerable social and economic significance for controlling fire risk in large cities.

Research on building fire risk assessment has been carried out worldwide for several decades. In the 1970s, the United States developed a risk index system that provided a structured basis for assessing fire risk and estimating potential fire losses [5]. In 1985, the United Kingdom issued what is widely regarded as the first major performance-based fire safety code [6]. From 1996 to 2002, four international symposia devoted to performance-based design codes and methods were convened [7]. During this period, three main tools for building fire risk assessment came into widespread use: the Swiss risk assessment method (SIA 81) [8], the Fire Risk Assessment Method for Engineering (FRAME) [9], and the Fire Risk Evaluation and Cost Assessment Model (FIRECAM) [10].

In building fire risk assessment, the fire risk index (FRI) approach is widely applied. Koutsomarkos et al. carried out a detailed review of the FRI based on existing studies [11], and Brzezińska et al. developed a method tailored to simulate the fire safety performance level of sustainable buildings [12]. Salazar et al. introduced the Potential Fire Loss Index, an index-based tool for the assessment of fire vulnerability in heritage buildings [13]. At the urban scale, Palazzi et al. used the fire risk index approach to assess fire risk in clusters of historic buildings within city districts [14]. Focusing on the safety performance of in-building fire-protection systems, Liu et al. developed a fire risk assessment framework for large commercial buildings [15]. Wang et al. proposed a fire risk assessment method for large commercial and high-rise buildings that integrates intuitionistic fuzzy sets with social network graphs [16]. Zou et al. evaluated fire risk in shopping malls using a quantitative safety checklist coupled with the structure entropy weight method [17]. Ketsakorn et al. assessed the fire risk of buildings in schools for the blind by combining a fire inspection checklist, brainstorming, and the analytic hierarchy process [18]. Ju et al. integrated a game-theoretic combined-weighting approach with the TOPSIS method to develop a risk-value model for metro stations [19]. With advances in computing, computer-based techniques have been increasingly applied to building fire risk assessment. Lei et al. proposed a machine-learning-based, fire risk-index-driven solution to predict the fire risk level of timber historic buildings [20]. Zhang et al. proposed an evaluation approach that integrates a spatial Markov chain model with an index-based system, using historical high-rise fire incident data to estimate the probability of fire occurrence in high-rise buildings [21]. Ferreira et al. developed a new urban fire risk assessment methodology, computed fire risk levels for more than 500 buildings within a city and performed spatial analysis of the results using integrated geographic information system (GIS) tools [22]. In addition, numerous researchers have examined and characterized the contributory fire risk factors within buildings. Salazar et al. reviewed existing index-based fire risk assessment methods and proposed a vulnerability index tailored to heritage buildings [23]. Dabous et al. classified and evaluated the effectiveness of fire-prevention and mitigation technologies for high-rise buildings [24]. Li et al. assessed fire risk in traditional vernacular dwellings using the fuzzy analytic hierarchy process in combination with the entropy weight method [25]. Beljikangarlou et al. developed a fire risk assessment approach to examine fire risk factors in hospitals [26].

Although existing studies have greatly advanced building fire risk assessment, certain limitations remain when these approaches are directly applied to high-rise building fire risk assessment [27]. First, many assessment frameworks mainly focus on fire hazards, building vulnerability, or the reliability of fire-protection facilities, whereas the complete chain of high-rise building fire risk formation has not been sufficiently represented. Second, high-rise buildings differ significantly from other building types in terms of spatial morphology, occupant composition, fire spread, and evacuation difficulty. Therefore, when constructing a fire risk assessment indicator system for high-rise buildings, it is necessary to incorporate risk factors that reflect the specific characteristics of high-rise buildings.

Therefore, this study takes the fire risk formation mechanism of high-rise buildings as the starting point and reconstructs the assessment indicator system within the framework of Integrated Disaster Risk Theory. On this basis, an assessment method combining indicator weighting and the cloud model is further developed to support weight allocation, uncertainty characterization, and risk level determination. The main contributions of this study are summarized as follows.

First, a fire risk assessment indicator system specifically applicable to high-rise buildings is constructed based on the Integrated Disaster Risk Theory. By conceptualizing high-rise building fires as a type of human-induced disaster, this study establishes an assessment framework from four dimensions: hazard, exposure, vulnerability, and emergency response and recovery capacity. Compared with general building or commercial-complex fire risk assessment frameworks, the proposed system further incorporates indicators that reflect the specific characteristics of high-rise buildings, such as occupant activity intensity, building thermal insulation and exterior wall decoration, evacuation facility capacity, tangible and intangible assets, refuge floors and refuge facilities, and fire-protection facilities. This indicator system can more comprehensively represent the formation mechanism of high-rise building fire risk, including fire occurrence, fire spread, occupant evacuation, potential losses, and damage-control conditions.

Second, the emergency response and recovery capacity dimension is strengthened according to the emergency-response characteristics of high-rise building fires. Considering the difficulties of vertical evacuation, rapid upward spread of fire and smoke, limited external fire-suppression conditions, and strong dependence on professional rescue forces, this study integrates both building-level internal response capacity and city-level external rescue capacity into the assessment framework. This design enables the assessment to better reflect the synergistic effect of internal initial response and external professional rescue in high-rise building fire emergencies.

## 2. Methods

Based on the Integrated Disaster Risk Theory, this study systematically constructed a high-rise building fire risk assessment indicator system from four dimensions and, on this basis, conducted a comprehensive fire risk assessment. The field investigation of some of the equipment involved in the case study is shown in Fig 1.

**Fig 1:**
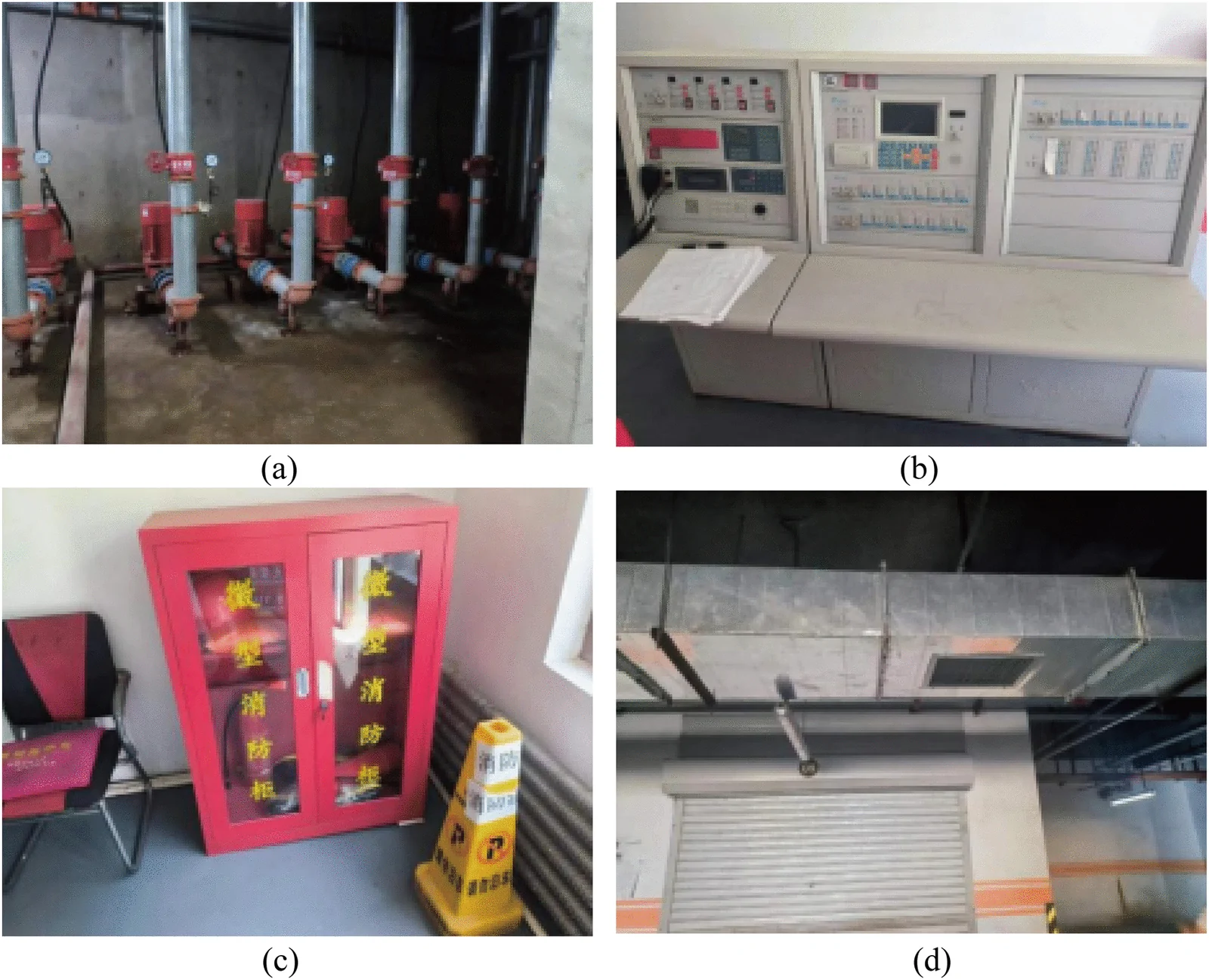
(a) fire pump for the fire service water supply; (b) fire control panel; (c) compact firefighting equipment cabinet; (d) ventilation duct. (Reprinted from a field photograph under a CC BY license, with permission from Jilin Shangbang Real Estate Development Co., Ltd., original copyright 2016).

This study was conducted in the following four steps:

Step 1: Construction of a high-rise building fire risk assessment indicator system.

First, data related to high-rise building fires, existing assessment methods, and fire-related influencing factors were systematically collected through field investigations, literature review, and interviews with third-party fire risk assessment agencies. Second, based on the Integrated Disaster Risk Theory, the formation mechanism of high-rise building fire risk was analyzed in depth from four dimensions, namely hazard (H), exposure (E), vulnerability (V), and emergency response and recovery capacity (C), and the key influencing factors were identified. Finally, with comprehensive consideration given to both reducing the probability of fire occurrence and improving fire suppression efficiency, and in combination with field conditions and data availability, a fire risk assessment indicator system applicable to high-rise buildings was constructed.

Step 2: Determination of indicator weights using a game-theoretic combination weighting method. In this study, the fuzzy analytic hierarchy process based on a fuzzy consistent matrix (FAHP) was employed to address the fuzzy judgment problem in the assessment process, while the structure entropy weight method (SEWM) was used to calculate indicator weights by integrating both subjective and objective information. On this basis, a game-theoretic combination weighting method was introduced to comprehensively optimize the weights obtained from these two methods, thereby reducing the bias that may arise from any single method and improving the rationality and stability of the weighting results.

Step 3: Development of a high-rise building fire risk assessment model. After the assessment indicators and their weights were determined, the cloud model was employed to address the fuzziness and randomness in the assessment process, and a high-rise building fire risk assessment model based on game-theoretic combination weighting and the cloud model was constructed. This model was used to comprehensively assess both the likelihood of fire occurrence and its potential consequences, thereby determining the fire risk level of the assessed object.

Step 4: Comparison with an established fire risk assessment method. To examine the plausibility and consistency of the comprehensive assessment model proposed in this study, a typical high-rise building in a residential community in Changchun was selected as a case study for fire risk scoring and risk level determination, and the assessment results were compared with those obtained using an existing well-established method [28]. The case study demonstrated that the model developed in this study showed good feasibility and consistency, thereby supporting its feasibility and consistency in practical assessment.

The overall research workflow of this study is illustrated in Fig 2.

**Fig 2:**
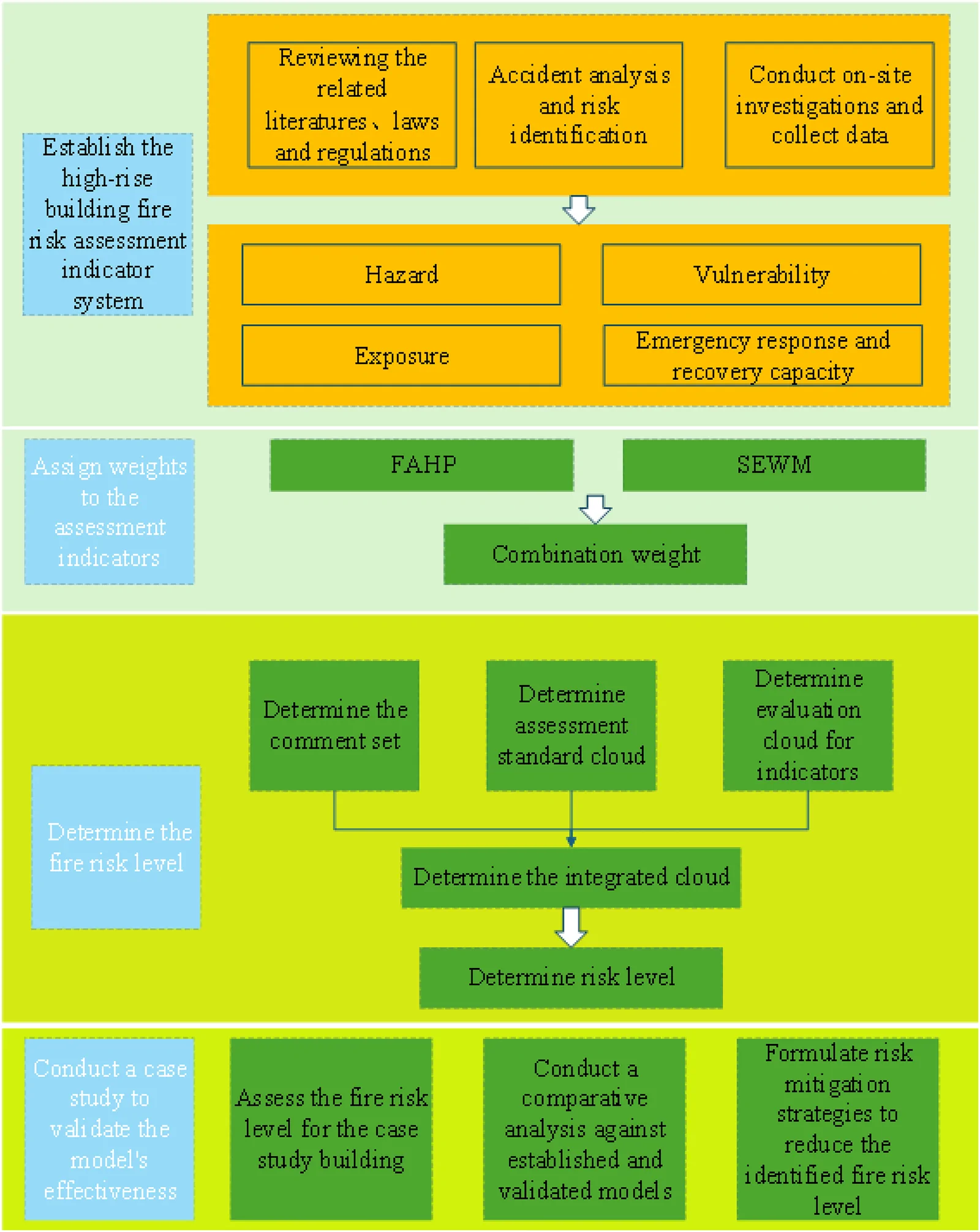
Research roadmap.

### 2.1. High-rise building fire risk assessment indicator system

Integrated Disaster Risk Theory characterizes disaster risk through four dimensions: hazard, exposure, vulnerability, and emergency response and recovery capacity [29,30]. As building fires are regarded as human-induced disasters [31], this framework was extended in this study to high-rise building fire risk assessment. Accordingly, the indicator system was constructed from these four dimensions while incorporating the specific characteristics of high-rise building fires, particularly vertical evacuation and emergency rescue requirements.

Hazard refers to the inherent variability of disaster-inducing factors and is mainly determined by the intensity and frequency of hazardous events. In this study, hazard primarily refers to the fire-inducing factors and fire environment associated with high-rise building fires. According to data released by China’s National Fire and Rescue Administration and the proceedings of the Second Building Fire Protection Conference [32], electrical faults and improper use of fire are the main causes of building fires. Therefore, occupant movement, electrical facilities, and gas facilities are selected in this study as important indicators characterizing fire-inducing factors. Specifically, occupant activity intensity (H_1_) reflects the frequency of human activities, the complexity of occupants’ use behaviors, and the possibility of fire occurrence caused by human factors such as careless use of fire and unauthorized use of electrical equipment in high-rise buildings. Electrical facilities (H_2_) reflect the fire risk caused by ageing electrical wiring, overloaded equipment operation, short-circuit faults, and inadequate maintenance of power distribution systems. Gas facilities (H_3_) reflect the possibility of fire accidents such as combustion and explosion caused by leakage, ageing, improper use, or inadequate maintenance of gas pipelines, valves, stoves, and related equipment. Meanwhile, the hazard of high-rise building fires is influenced not only by ignition-inducing factors, but also by the internal and external fire environment of the building. Therefore, external environment, internal fire load, building insulation and exterior wall decoration are further selected as hazard assessment indicators. Among them, external environment (H_4_) reflects the influence of surrounding hazardous sources, fire spread from adjacent buildings, wind conditions, and the distribution of external combustibles on fire occurrence and development. Indoor fire load (H_5_) reflects the influence of the quantity, type, and calorific density of combustibles in residential, commercial, office, storage, and other functional spaces on fire intensity and fire spread rate. Building thermal insulation and exterior wall decoration (H_6_) reflect the combustion performance of exterior wall insulation materials and decorative materials, as well as their hazard potential in façade fire spread, smoke diffusion, and fire escalation.

Exposure refers to all people and property that may be affected by hazardous factors and arises from the interaction between disaster-inducing factors and disaster-bearing bodies. In high-rise building fires, exposure mainly reflects the potential casualties and economic losses that may occur after a fire breaks out. Because high-rise buildings are usually characterized by high occupant density, mixed functions, long vertical evacuation distances, and complex spatial organization, a larger building population and more frequent occupant activities may lead to a higher likelihood of casualties and evacuation congestion after a fire occurs. Meanwhile, the more complex the building functions are, the wider the potential impact range of fire becomes, and the interaction among different functional spaces may further increase the difficulty of risk propagation and emergency response. Therefore, this study selects five indicators from four aspects, namely human exposure, functional exposure, evacuation exposure, and asset exposure, to characterize the exposure of high-rise building fires. Specifically, population size within the building (E_1_) reflects the number of people who may be threatened during a fire and the associated potential casualty risk. Building functional complexity (E_2_) reflects the fire impact range and the degree of risk coupling under mixed-use conditions involving residential, office, commercial, catering, storage, and other functions. Evacuation facility capacity (E_3_) reflects the capacity of evacuation facilities, such as stairwells, evacuation corridors, and safety exits, to meet the evacuation demand of occupants in the building, thereby indicating evacuation pressure and passage congestion risk under fire scenarios. Tangible assets of the building (E4) reflect the direct economic losses that may be caused to the building structure, equipment and facilities, and indoor property under the effects of fire, smoke, and firefighting water. This indicator was evaluated by considering the economic value of the building, including property value, construction cost, building scale, and potential direct asset losses caused by fire. Intangible assets of the building (E5) reflect indirect losses caused by fire, including business interruption, production suspension, loss of service functions, social impacts, and recovery costs. For the residential building investigated in this study, this indicator was evaluated by considering the interruption of residential functions, impacts on ground-floor commercial services, social impacts, recovery requirements, and potential changes in property value after similar fire events.

Vulnerability is an inherent damage-prone attribute of disaster-bearing bodies, reflecting both the likelihood that they will suffer damage under the impact of a disaster and their capacity to resist disaster impacts. In the formation process of high-rise building fire risk, vulnerability mainly refers to the tendency of disaster-bearing bodies to experience casualties, structural damage, and functional failure under the effects of fire, high-temperature smoke, and constrained external rescue conditions. Compared with ordinary buildings, high-rise buildings are characterized by greater vertical height, longer evacuation routes, more complex occupant composition, and greater difficulty in fire suppression. Therefore, their vulnerability is reflected not only in the fire resistance and fire-protection performance of the building itself, but also in the emergency response and self-evacuation capabilities of occupants. In this study, the disaster-bearing bodies involved in high-rise building fires include both occupants and the building itself. In terms of human vulnerability, older adults and children generally have weaker risk perception, mobility, and emergency response capabilities during fires [33,34]. They are also more likely to be affected by smoke, high temperatures, and crowded conditions during evacuation. Therefore, the proportion of elderly and children is adopted as an indicator for measuring human vulnerability. In terms of building vulnerability, according to current fire safety codes [35,36], a building’s capacity to resist fire damage mainly depends on its fire resistance performance, fire compartmentation, evacuation conditions, and the configuration level of fire-protection facilities. Accordingly, this study selects indicators including building height, building service life, fire compartmentation, fire resistance rating, evacuation routes and evacuation guidance facilities, water-supply facilities, automatic sprinkler system, automatic fire alarm system, and ventilation and smoke exhaust system to characterize the fire resistance, emergency avoidance, and loss-reduction capabilities of high-rise buildings under fire conditions.

Emergency response and recovery capacity refers to the comprehensive capacity of disaster-affected objects to conduct risk prevention, initial response, emergency rescue, and post-disaster recovery before and after a fire occurs. It encompasses emergency management capability, mitigation investment, resource reserves, and related aspects. In the formation process of high-rise building fire risk, this capacity not only affects hazard control and risk prevention before a fire occurs, but is also directly related to initial fire suppression, occupant evacuation, fire spread control, and post-disaster recovery after a fire breaks out. Because high-rise buildings are characterized by difficult vertical evacuation, rapid upward spread of fire and smoke through vertical spaces, great difficulty in external fire suppression, and strong dependence on professional fire and rescue forces, assessing emergency response and recovery capacity solely from the perspectives of internal fire safety management and facility maintenance cannot fully reflect the practical requirements of emergency response in high-rise building fires. Therefore, this study considers both the internal initial response capacity of the building and the city-level external fire and rescue capacity within the dimension of emergency response and recovery capacity. On the one hand, internal response capacity is mainly characterized by indicators such as fire safety management, fire-fighting facility maintenance, and the construction status of micro fire stations, which reflect the building’s own capacity for routine prevention and control, hazard identification, facility support, and initial emergency response. On the other hand, external rescue capacity is mainly characterized by indicators such as the comprehensive rescue capability of the jurisdictional fire and rescue station, fire response time, and the compatibility between fire-fighting equipment and building characteristics, which reflect the arrival speed of professional fire forces, the allocation level of fire suppression and rescue resources, and their adaptability to complex high-rise building fire scenarios.

The fire risk assessment **indicator** system is shown in Table 1.

**Table 1:** High-rise building fire risk assessment indicator system.

HRFRI	Factor	Index
High-rise building fire risk assessment indicator system	Hazard (H)	Occupant activity intensity (H_1_)
		Electrical facilities (H_2_)
		Gas facilities (H_3_)
		External environment (H_4_)
		Indoor fire load (H_5_)
		Building thermal insulation and exterior wall decoration (H_6_)
	Exposure (E)	Population size (E_1_)
		Building functional complexity (E_2_)
		Evacuation facility capacity (E_3_)
		Tangible assets of the building (E_4_)
		Intangible assets of the building (E_5_)
	Vulnerability (V)	Proportion of elderly and children (V_1_)
		Building height (V_2_)
		Building service life (V_3_)
		Fire compartment (V_4_)
		Fire-resistance rating (V_5_)
		Evacuation routes and evacuation guidance facilities (V_6_)
		Refuge floors and refuge facilities (V_7_)
		Water-supply facilities (V_8_)
		Automatic fire alarm system (V_9_)
		Automatic sprinkler system (V_10_)
		Ventilation and smoke exhaust system (V_11_)
	Emergency response and recovery capacity (C)	Fire management system (C_1_)
		Fire-safety inspection (C_2_)
		Fire-facility maintenance (C_3_)
		Construction status of micro fire stations (C_4_)
		Comprehensive rescue capacity of the local fire station (C_5_)
		Firefighting response time (C_6_)
		Compatibility between firefighting equipment and building characteristics (C_7_)

### 2.2. Determination of index weights

#### 2.2.1. The Fuzzy Analytic Hierarchy Process.

FAHP is a multi-criteria decision-making method that integrates fuzzy set theory with the traditional analytic hierarchy process (AHP). By combining hierarchical structuring with fuzzy mathematics, FAHP can accommodate uncertainty and ambiguity in expert judgments, thereby enhancing the reliability and effectiveness of group decision-making [37,38]. The main steps of FAHP in this study were as follows:

1. Construct the fuzzy-complementary judgment matrix A.

Using the 0.1–0.9 scaling method, we performed pairwise comparisons among the factors to obtain the fuzzy judgment matrix $A=(a_{ij}{)}_{n\times n}$. Detailed descriptions of the scale levels are provided in Table 2.

**Table 2:** Pairwise comparison scale based on the 0.1–0.9 method.

Scale	Define	Description
0.5	Just as important	Comparing $a_{i}$and $a_{j}$, they are equally important.
0.6	Slightly more important	Comparing $a_{i}$and $a_{j}$, $a_{i}$ is slightly more important than $a_{j}$.
0.7	More important	Comparing $a_{i}$and $a_{j}\;$, $a_{i}\;$is obviously more important than $a_{j}$.
0.8	Very important	Comparing $a_{i}$and $a_{j}$, $a_{i}$ is much more important than $a_{j}$.
0.9	Absolutely important	Comparing $a_{i}$and $a_{j}$, $a_{i}\;$is absolutely more important than $a_{j}$.
0.1,0.2,0.3,0.4	Inverse comparison	If the element $a_{i}$ is compared with the element $a_{j}$ to obtain the judgment $r_{ij}$, then the element $a_{j}$ is compared with the element $a_{i}$ to be judged as$r_{ji}=\text{1}-r_{ij}$

2. Calculation of the weight vector W

$$\begin{array}{c} \begin{array}{c} \;W_{i}=\frac{\sum_{j=\text{1}}^{n}a_{ij}+\frac{n}{\text{2}}-\text{1}}{n(n-\text{1})},i=\text{1},\text{2},\cdot \cdot \cdot ,n \end{array} \end{array}$$ (1)

where $\sum_{j=\text{1}}^{n}a_{ij}\;$denotes the sum of the elements in the i-th row.

3. Calculation of the characteristic matrix$W^{*}$

Let $W=(W_{\text{1}},W_{\text{2}},\cdot \cdot \cdot ,W_{n}{)}^{T}$ denote the weight vector of the fuzzy judgment matrix A, where $\sum_{i=\text{1}}^{n}W_{i}=\text{1},W_{i}\geq \text{0}(i=\text{1},\text{2},\cdot \cdot \cdot ,n)$, and define:


$$\begin{array}{c} \begin{array}{c} {\;W}_{ij}=\frac{W_{i}}{W_{i}+W_{j}}(\forall i,j=\text{1},\text{2},\cdot \cdot \cdot ,n) \end{array} \end{array}$$ (2)

For example, the element $W_{\text{01}}$ is given by $\frac{W_{\text{0}}}{W_{\text{0}}+W_{\text{1}}}$. By applying this computation rule to every position of the n×n matrix, using the corresponding components of the weight vector W, an n-th-order matrix is obtained:


$$\begin{array}{c} \begin{array}{c} W^{*}=(W_{ij}{)}_{n\times n} \end{array} \end{array}$$ (3)

$W^{*}$ is referred to as the characteristic matrix of the judgment matrix A.

4. Calculation of the compatibility indexI

Let $A=(a_{ij}{)}_{n\times n}$and $B=(b_{ij}{)}_{n\times n}$ be two fuzzy judgment matrices, and define:


$$\begin{array}{c} \begin{array}{c} I(A,B)=\frac{\text{1}}{n^{\text{2}}}\sum_{i=\text{1}}^{n}\sum_{j=\text{1}}^{n}\;\left|a_{ij}+b_{ji}-\text{1}\right| \end{array} \end{array}$$ (4)

as the compatibility index between A and B.

5. Consistency test

The compatibility index between the fuzzy judgment matrix A and its characteristic matrix $W^{*}$, denoted $I(A,W^{*})$, is computed. If $I(A,W^{*})\leq \alpha$, the consistency test is satisfied.

A smaller value of α indicates a stricter consistency requirement for the fuzzy judgment matrix. In practical applications, α=0.1 is commonly adopted.

#### 2.2.2. The structure entropy weight method.

The structure entropy weight method combines subjective and objective weighting, effectively reducing the uncertainty that may arise during index ranking and improving the accuracy of expert-assigned weights [39]. The specific steps of this method in this study were as follows:

1. Expert opinions were collected to construct a representative ranking matrix.

Based on the Delphi method, importance-ranking information for the index was collected from several experts in the relevant field. All experts were randomly divided into groups, and each group independently ranked the importance of all indices, thereby forming a set of representative ranking matrices.

The indices were ranked in descending order of importance. For example, a label of “1” denotes “most important”, “2” denotes “more important”, “3” denotes “important”, and so on. If certain indices were considered equally important, they could be assigned to the same label.

Suppose there are *ζ* expert groups providing important rankings for the index. Each group has a scoring-index set $C\;=\;(\;c_{\text{1}},c_{\text{2}},\ldots ,c_{n}\;)$, and the corresponding typical ranking array for this set is denoted by $\left(\;a_{i\text{1}},\;a_{i\text{2}},\ldots ,\;a_{in}\right)$*.*

Then ζ typical ranking matrices were obtained, denoted as$\;A={\left(a_{ij}\right)}_{\zeta \;\times \;n},i=\text{1},\text{2},\ldots ,\zeta ;j=\text{1},\text{2},\ldots ,n$, where ${\text{a}}_{\text{ij}}$ represents the assessment score assigned by the i-th expert group to the j-th index $\;c_{j}$.

The expert opinion collection form is presented in Table 3.

**Table 3:** Expert opinion collection form.

	Index 1	Index 2	Index 3	Index 4	Index 5	...
**Expert 1**						
**Expert 2**						
**Expert 3**						
**Expert 4**						
**...**

2. A blind analysis was conducted using the entropy method to calculate the overall degree of consensus.

An entropy analysis was performed on the typical ranking matrix obtained in Step 1, and the transformed entropy function is defined as


$$\begin{array}{c} \begin{array}{c} \;\mu (I)=\frac{ln\left(m-I\right)}{ln\left(m-\text{1}\right)} \end{array} \end{array}$$ (5)

In Eq. (5), I denotes the importance-ranking value assigned by an expert group to a given index, and m is the transformation parameter.

Let m=J+2, where J is the maximum ranking value. Substituting each $I=a_{ij}\;$from the typical ranking matrix A into Eq. (5) yields a quantitative transformation of $a_{ij}$; by setting $c(a_{ij})=b_{ij}$, the membership matrix of the ranking scores is obtained as$\;B\;={\left(b_{ij}\right)}_{\zeta \times \;n},i\;=\;\text{1},\;\text{2},\ldots ,\;\zeta ;\;j\;=\;\text{1},\;\text{2},\ldots ,\;n.$

Assuming each expert group has equal weight for index $c_{j}$(i.e., “consistent views”), the average recognition degree $b_{j}\;$is defined as


$$\begin{array}{c} \begin{array}{c} b_{j}=\frac{b_{\text{1}j}+b_{\text{2}j}+....+b_{\zeta j}}{\zeta } \end{array} \end{array}$$ (6)

The cognition-induced uncertainty of expert group i regarding index $c_{j}\;$is defined as the recognition blindness $Q_{j}$, which is expressed as


$$\begin{array}{c} \begin{array}{c} Q_{j}=\left\{\frac{\left[max(b_{\text{1}j},b_{\text{2}j},....,b_{\zeta j})-b_{j}\right]+\left[b_{j}-min(b_{\text{1}j},b_{\text{2}j},....,b_{\zeta j})\right]}{\text{2}}\right\} \end{array} \end{array}$$ (7)

For each index $c_{j}$, the overall recognition degree of the ζ expert groups regarding $c_{j}\;$is defined as


$$\begin{array}{c} \begin{array}{c} k_{j}=b_{j}(\text{1}-Q_{j}) \end{array} \end{array}$$ (8)

Collecting the values $k_{j}\;$over all indices yields the assessment vector$K=(k_{\text{1}},k_{\text{2}},\cdot \cdot \cdot ,k_{n})$

3. Normalization and determination of the comprehensive index weights.

The assessment vector K was normalized as follows:


$$\begin{array}{c} \begin{array}{c} \omega_{j}=\frac{k_{j}}{\sum_{j=\text{1}}^{n}k_{j}} \end{array} \end{array}$$ (9)

The normalized result $\omega_{j}$ is thus taken as the weight of each index $c_{j}\;$obtained by the structure entropy weight method.

#### 2.2.3. Game-theoretic combination weighting.

To enhance the scientific soundness of index weighting, this study adopted a game-theoretic combination weighting approach based on the weights derived from FAHP and SEWM, thereby determining the final weights of all assessment indices.

The fundamental idea of game-theoretic combination weighting is to seek consistency and compromise among the different weights assigned to a given assessment index by minimizing the deviation between the combined weight and each of the individual basic weight vectors. This approach helps enhance the reliability of the assessment results [40,41]. The calculation steps of this approach were as follows:

1. Using L different methods, we first obtained the weights of the n indices as

$$\begin{array}{c} \begin{array}{c} w_{l}\;=(w_{l\text{1}},w_{l\text{2}},\cdots ,w_{ln}),\;l=\text{1},\text{2},\cdots ,L \end{array} \end{array}$$ (10)

The linear combination of these L weight vectors can be expressed as


$$\begin{array}{c} \begin{array}{c} \;\text{w}=\sum_{\text{l}=\text{1}}^{\text{L}}\alpha_{\text{l}}{\text{w}}_{\text{l}}^{\text{T}}\;(\alpha_{\text{l}}>\text{0}) \end{array} \end{array}$$ (11)

where w is the combined weight vector and $\alpha_{l}$ denotes the linear-combination coefficient corresponding to the l-th method.

2. Based on the game-theoretic combined weighting principle, the deviation between the combined weight vector w and each basic weight vector $w_{p}$is minimized. The objective function is given by

$$\begin{array}{c} \begin{array}{c} \text{min}{\left\|\sum_{l=\text{1}}^{L}\alpha_{l}w_{l}^{T}-w_{p}^{T}\right\|}_{\text{2}},\;p=\text{1},\text{2},\cdots ,L \end{array} \end{array}$$ (12)

3. According to the properties of matrix differentiation, the first-order optimality condition of the above optimization problem can be written as:

$$\begin{array}{c} \begin{array}{c} \sum_{l=\text{1}}^{L}\alpha_{l}w_{p}w_{l}^{T}=w_{p}w_{p}^{T},\;p=\text{1},\text{2},\cdots ,L \end{array} \end{array}$$ (13)

which is equivalent to the following linear system:


$$\begin{array}{c} \begin{array}{c} \;[\begin{array}{cccc} w_{\text{1}}w_{\text{1}}^{T} & w_{\text{1}}w_{\text{2}}^{T} & \cdots & w_{\text{1}}w_{L}^{T}\\ w_{\text{2}}w_{\text{1}}^{T} & w_{\text{2}}w_{\text{2}}^{T} & \cdots & w_{\text{2}}w_{L}^{T}\\ \vdots & \vdots & \vdots & \vdots \\ w_{L}w_{\text{1}}^{T} & w_{L}w_{\text{2}}^{T} & \cdots & w_{L}w_{L}^{T} \end{array}]\left[\begin{array}{c} \alpha_{\text{1}}\\ \alpha_{\text{2}}\\ \vdots \\ \alpha_{L} \end{array}\right]=\left[\begin{array}{c} w_{\text{1}}w_{\text{1}}^{T}\\ w_{\text{2}}w_{\text{2}}^{T}\\ \vdots \\ w_{L}w_{L}^{T} \end{array}\right] \end{array} \end{array}$$ (14)

4. Solving the linear system in Eq. (14) yields the optimal combination coefficients $\left(\alpha_{\text{1}},\alpha_{\text{2}},\ldots ,\alpha_{L}\right)$, which are then normalized. Since some coefficients may be negative, their absolute values are taken prior to normalization:

$$\begin{array}{c} \begin{array}{c} \;{\;\alpha }_{l}^{*}=\frac{\left|\alpha_{l}\right|}{\sum_{l=\text{1}}^{L}|\alpha_{l}|} \end{array} \end{array}$$ (15)

5. Finally, the combined weight vector is obtained as

$$\begin{array}{c} \begin{array}{c} {\;w}^{*}=\sum_{l=\text{1}}^{L}\alpha_{l}^{*}w_{l}^{T},\;l=\text{1},\text{2},\cdots ,L \end{array} \end{array}$$ (16)

### 2.3. Comprehensive assessment based on the cloud model

#### 2.3.1. Basic concepts of the cloud model.

Cloud model theory, proposed by Academician Deyi Li based on probability theory and fuzzy mathematics, is a theory that reflects the fuzziness and randomness of concepts in human knowledge systems and investigates the transformation between uncertain information and quantitative indicators. It can effectively overcome the limitation of traditional fuzzy assessment methods, in which concepts cannot be quantitatively represented, thereby improving assessment accuracy.

Definition of a cloud drop.

Let U be the universe of discourse of quantitative data, and let C be a qualitative concept defined on U, for example x∈U is a random realization of the qualitative concept C in U, and the degree of association of x with U denoted by μ(x)∈[0,1] is a random number with a stable tendency:


$$\begin{array}{c} \begin{array}{c} \mu :U\to [\text{0},\text{1}]\;\forall x\in U\;x\to \mu (x) \end{array} \end{array}$$ (17)

then the distribution of the quantitative value x over the universe of discourse U is called a cloud, and each x is referred to as a cloud drop.

Digital characteristics of the cloud

The digital characteristics of a cloud are described by three parameters, namely Ex,En,He. Among them, the expected value Ex (Expected Value) represents the expected position of cloud drops in the universe of discourse and serves as the central value of the concept in the discourse domain. The entropy En (Entropy) denotes a measure of the uncertainty of the qualitative concept, reflecting the dispersion degree of cloud drops in the universe of discourse and used to characterize the span of the cloud. The hyper-entropy He (Hyper Entropy) represents the uncertainty measure of entropy, reflecting both the dispersion of cloud drops and the randomness of certainty, and is used to characterize the thickness of the cloud.

The cloud model employs cloud generators (Fig 3) to establish the mapping relationship between qualitative concepts and quantitative representations.

**Fig 3:**
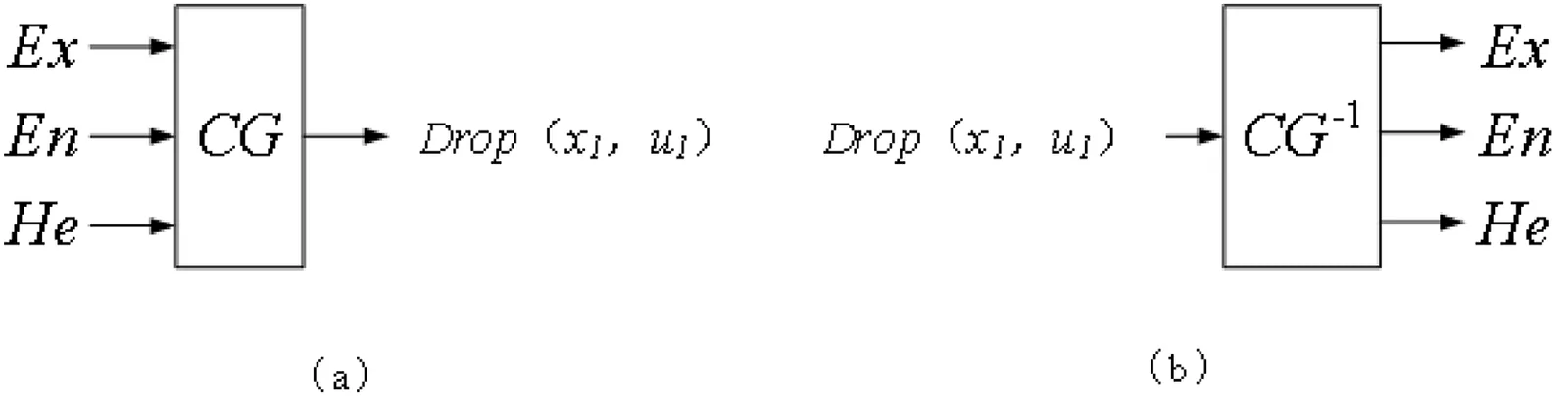
(a) Forward cloud generator; (b) Backward cloud generator.

#### 2.3.2. Comprehensive assessment based on the cloud model.

1. Determination of the comment set.

The comment set refers to the set of all possible assessment results that evaluators may assign to the object being assessed. After the comment set is determined, an interval range should also be defined for each assessment grade.

2. Determination of the standard cloud.

The standard cloud digital characteristic values corresponding to the i-th evaluation interval, the standard cloud digital characteristic values $\left[x_{i}^{min},{\;x}_{i}^{max}\right]$ are $\left(Ex_{i},En_{i},He\right)$,the calculation formulas are as follows:


$$\begin{array}{c} \begin{array}{c} \;\left\{ \begin{array}{cc} & Ex_{i}=\left(x_{i}^{\text{max}}+x_{i}^{\text{min}}\right)\;/\;\text{2}\\ & En_{i}=\left(x_{i}^{\text{max}}-x_{i}^{\text{min}}\right)\;/\;\text{6}\\ & He=k \end{array}\;\right.\; \end{array} \end{array}$$ (18)

In the formula k is a constant, which is taken as 0.5 in this paper.

3. Determination of the indicator evaluation cloud.

Questionnaires on indicator evaluation were collected from experts, with each column representing an indicator and each row representing an expert, thereby forming an evaluation matrix. Assuming that there are m experts and n indicators, the dimension of the evaluation matrix Z is m×n. The evaluation matrix is expressed as follows, where $z_{ij}$ denotes the evaluation value assigned by expert i to indicator j.


$$\begin{array}{c} \begin{array}{c} Z=[\begin{array}{cccc} z_{\text{1}\text{1}} & z_{\text{1}\text{2}} & \ldots & z_{\text{1}n}\\ z_{\text{2}\text{1}} & z_{\text{2}\text{2}} & \ldots & z_{\text{2}n}\\ \ldots & \ldots & \ldots & \ldots \\ z_{m\text{1}} & z_{m\text{2}} & \ldots & z_{mn} \end{array}] \end{array} \end{array}$$ (19)

Then, the backward cloud generator was employed to calculate the digital characteristics of the scores assigned by all experts for each indicator, namely $C_{j}\left(Ex_{j},En_{j},He_{j}\right)$,In this study, the SBCT-1stM algorithm was employed


$$\begin{array}{c} \begin{array}{c} \left\{ \begin{array}{cc} & Ex_{j}=\frac{\text{1}}{N}\sum_{i=\text{1}}^{N}\;z_{ij}\\ & En_{j}=\sqrt{\frac{\pi }{\text{2}}}\times \frac{\text{1}}{N}\sum_{i=\text{1}}^{N}\;\left|z_{ij}-Ex_{j}\right|\\ & S_{j}^{\text{2}}=\frac{\text{1}}{N-\text{1}}\sum_{i=\text{1}}^{N}\;{\left(z_{ij}-Ex_{j}\right)}^{\text{2}}\\ & He_{j}=\sqrt{\left|S_{j}^{\text{2}}-En_{j}^{\text{2}}\right|} \end{array}\right.\; \end{array} \end{array}$$ (20)

4. Calculation of the comprehensive evaluation cloud.

By combining the indicator weights $w_{j}$ with the following formulas, the digital characteristics of the comprehensive evaluation cloud can be obtained:


$$\begin{array}{c} \begin{array}{c} \left\{ \begin{array}{cc} & Ex=\sum_{j=\text{1}}^{n}\;\left(Ex_{j}\cdot w_{j}\right)\\ & En=\sqrt{\sum_{j=\text{1}}^{n}\;\left(En_{j}^{\text{2}}\cdot w_{j}\right)}\\ & He=\sum_{j=\text{1}}^{n}\;\left(He_{j}\cdot w_{j}\right) \end{array}\right.\; \end{array} \end{array}$$ (21)

5. Calculation of cloud similarity

Let the digital characteristics of the comprehensive evaluation cloud be denoted as $C_{\text{1}}(Ex_{\text{1}},En_{\text{1}},He_{\text{1}})$, and those of a standard evaluation cloud be denoted as $C_{\text{2}}(Ex_{\text{2}},En_{\text{2}},He_{\text{2}})$, The procedure for calculating the similarity is as follows:

Generate a normal random variable $En=G(En_{\text{1}},He_{\text{1}})$, where $En_{\text{1}}$ denotes the expectation and $He_{\text{1}}$ denotes the standard deviation.

Generate a Normal random variable $x_{k}=G(Ex_{\text{1}},En)$, where $Ex_{\text{1}}$ denotes the expectation and En denotes the standard deviation.

③ Calculate the degree of membership $\mu_{k}$:


$$\begin{array}{c} \begin{array}{c} \mu_{k}=exp\left(-\frac{(x_{k}-Ex_{\text{2}}{)}^{\text{2}}}{\text{2}En_{\text{2}}^{\text{2}}}\right) \end{array} \end{array}$$ (22)

④ Repeat steps ①-③ above until n values of $\mu_{k}$ are generated and then calculate the similarity $\delta_{i}$.


$$\begin{array}{c} \begin{array}{c} \delta_{i}=\frac{\text{1}}{n}\sum_{k=\text{1}}^{n}\;\mu_{k} \end{array} \end{array}$$ (23)

6. Comprehensive evaluation

The similarity between the comprehensive evaluation cloud and each standard evaluation cloud is calculated. Then, based on the principle of maximum degree of membership, the comprehensive evaluation result is determined, and the evaluation level corresponding to the highest similarity is taken as the final evaluation grade.

## 3. Results

### 3.1. Case study

This study takes a typical high-rise building in Changchun City as an example. The building was officially completed on November 17, 2015, and sales began on January 1, 2016. The building features a cast-in-place reinforced-concrete structural system with a frame–shear wall configuration. The superstructure consists of 17 above-grade floors, with a total height of 49.15 m and no basement. It has a fire-resistance rating of Class II and is categorized as a Category II high-rise building. The designed service life of the building is 50 years. The building has a footprint of 929.62 m² and a gross floor area of 8,740.30 m². It houses approximately 500 households, with a total of around 1,700 permanent residents, more than half of whom are either aged 60 and above or under 14 years. There are no hazardous buildings in the vicinity, and the straight-line distance from the building to the local fire brigade is less than 50 m. Regarding occupancy, the ground floor contains commercial service outlets, the second floor accommodates both commercial outlets and residential units, and floors 3–17 are designated for residential use.

### 3.2. Calculation results

Before applying the cloud model to determine the fire risk level of the case-study high-rise building, all assessment indicators were first quantified, and the corresponding weights were calculated using FAHP, SEWM, and the game-theoretic combination weighting method. The detailed calculation procedures and results are provided as supplementary tables in the Supporting Information (Appendix Tables A1–A10), while the final combined weights are summarized in Table 4. Subsequently, cloud model theory was introduced to construct a fire risk assessment model for high-rise buildings. Based on the field investigation results, 12 experts from relevant fields were invited to score the assessment indicators. All invited experts have more than three years of professional experience in fire safety, emergency management, or building fire-protection operations. The expert panel consisted of three university researchers specializing in safety-related fields, three frontline firefighters from fire and rescue departments, three professionals from third-party fire safety institutions, and three fire-facility operators from property management companies. Before scoring, the experts were provided with the definition of each indicator and the general risk-level intervals presented in Table 5. No separate indicator-specific numerical anchoring criteria were prescribed. Each expert independently evaluated the indicators based on the actual conditions of the case-study building, the indicator definitions, the general risk-level intervals, and their professional knowledge. The individual scores were then directly used as inputs for the cloud model to characterize the uncertainty and fuzziness of expert judgments.

**Table 4:** Weights of all indicators.

Primary indicators				Secondary indicators				Overall weight
Indicator	FAHP	SEWM	Combined weight	Indicator	FAHP	SEWM	Combined weight	
H	0.3	0.3484	0.3311	H_1_	0.19	0.2076	0.2021	0.0669
				H_2_	0.1733	0.1837	0.1805	0.0598
				H_3_	0.15	0.1380	0.1417	0.0469
				H_4_	0.1333	0.1013	0.1113	0.0369
				H_5_	0.1833	0.2034	0.1971	0.0653
				H_6_	0.17	0.1660	0.1672	0.0554
E	0.2	0.1638	0.1767	E_1_	0.24	0.2850	0.2709	0.0479
				E_2_	0.165	0.1212	0.1348	0.0238
				E_3_	0.215	0.2354	0.229	0.0405
				E_4_	0.21	0.2102	0.2102	0.0371
				E_5_	0.17	0.1483	0.1551	0.0274
V	0.2667	0.2778	0.2738	V_1_	0.0927	0.0960	0.095	0.0260
				V_2_	0.0991	0.1191	0.1128	0.0309
				V_3_	0.0964	0.1061	0.1031	0.0282
				V_4_	0.0973	0.1168	0.1107	0.0303
				V_5_	0.1	0.1226	0.1156	0.0317
				V_6_	0.0918	0.0907	0.091	0.0249
				V_7_	0.09	0.0830	0.0852	0.0233
				V_8_	0.0945	0.1001	0.0984	0.0269
				V_9_	0.0791	0.0539	0.0617	0.0169
				V_10_	0.0827	0.0633	0.0694	0.0190
				V_11_	0.0764	0.0484	0.0572	0.0157
C	0.2333	0.2100	0.2183	C_1_	0.1214	0.0927	0.1016	0.0222
				C_2_	0.1381	0.1248	0.1289	0.0281
				C_3_	0.1571	0.1792	0.1723	0.0376
				C_4_	0.1238	0.0978	0.1059	0.0231
				C_5_	0.1405	0.1372	0.1383	0.0302
				C_6_	0.1714	0.2118	0.1992	0.0435
				C_7_	0.1476	0.1565	0.1537	0.0336

**Table 5:** Value range for each assessment level.

Risk level	Score range	Basic characteristics
Ⅰ	90-100	Fire risk is very low
Ⅱ	70-90	Fire risk is lower
Ⅲ	50-70	Fire risk is moderate
Ⅳ	30-50	Fire risk is higher
Ⅴ	0-30	Fire risk is very high

#### 3.2.1. Calculation of the standard clouds.

In this study, the fire risk levels of high-rise buildings were classified into five grades, from Grade I to Grade V. The corresponding value ranges for each grade were 100–90, 90–70, 70–50, 50–30, and 30–0, respectively (Table 5). According to Equation (18), the standard clouds were calculated, and the standard cloud map was then generated using MATLAB, as shown in Fig 4.

**Fig 4:**
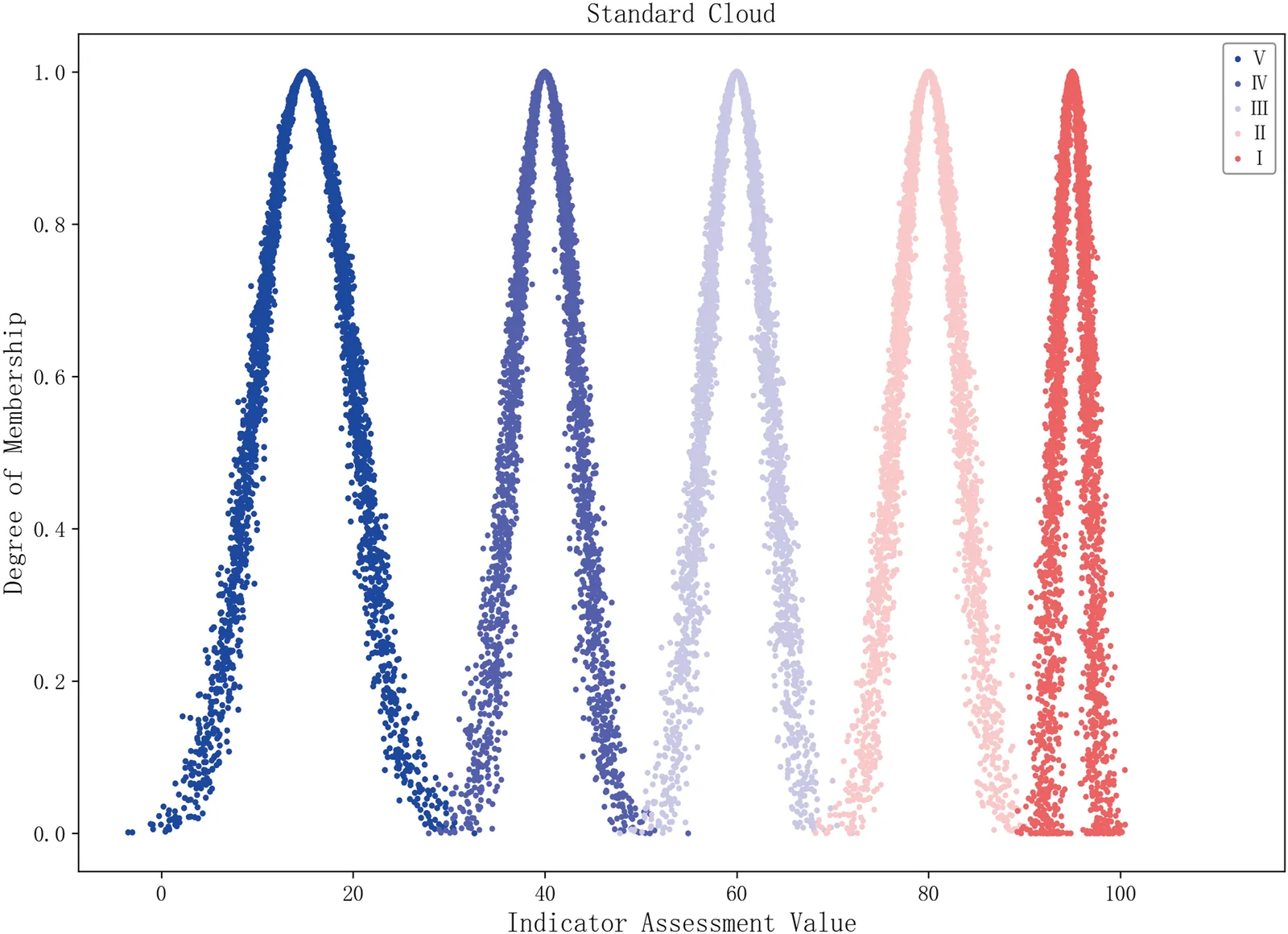
The standard cloud.

#### 3.2.2. Calculation of the assessment cloud.

Based on the actual conditions of the case-study high-rise building, 12 experts were invited to evaluate and score the assessment indicators. The assessment cloud was then calculated based on the evaluation results, and the calculated digital characteristics are presented in Table 6.

**Table 6:** Expert assessment cloud data.

Index	Score	Ex	En	He
H1	77,83,78,75,80,67,80,73,80,75,82,70	76.6667	4.874	0.2427
H2	83,81,80,80,80,77,72,82,83,75,78,86	79.75	3.5511	1.3993
H3	75,73,81,76,75,68,78,75,71,78,66,73	74.0833	4.0559	1.278
H4	86,90,84,95,88,80,88,88,94,83,77,89	86.8333	5.0481	1.4571
H5	67,80,68,77,81,76,80,67,73,79,72,68	74	6.0577	2.5163
H6	83,83,77,75,81,74,79,80,82,77,86,73	79.1667	4.1777	1.0583
E1	60,80,74,67,63,75,71,71,70,63,71,73	69.8333	5.5007	1.562
E2	66,74,74,73,78,72,79,77,88,74,75,74	75.3333	4.317	2.8551
E3	81,85,77,77,81,71,75,69,83,72,76,96	78.5833	6.9106	2.4788
E4	69,74,73,79,77,66,78,86,68,74,72,80	74.6667	5.5703	1.102
E5	82,87,75,81,87,82,83,79,84,75,78,79	81	3.9688	0.4984
V1	68,71,70,68,61,60,74,75,70,67,70,77	69.25	4.6477	2.0144
V2	84,78,76,77,80,64,76,79,82,78,73,75	76.8333	4.2125	2.7711
V3	86,83,86,87,86,88,86,80,83,77,89,83	84.5	3.4466	0.173
V4	81,81,78,80,84,77,81,75,83,73,83,74	79.1667	3.934	1.2995
V5	79,81,75,72,76,76,78,80,72,81,81,73	77	3.7599	1.3983
V6	85,86,74,78,80,80,72,82,83,67,75,77	78.25	5.5355	0.8087
V7	68,65,68,70,73,69,68,60,67,61,66,75	67.5	3.8644	1.8765
V8	83,85,76,81,79,80,77,76,78,80,85,85	80.4167	3.5336	0.8758
V9	82,87,76,82,82,82,84,84,84,83,88,91	83.75	3.2377	1.8814
V10	79,78,78,70,79,73,72,73,86,74,74,78	76.1667	4.3866	0.6029
V11	78,82,74,77,76,75,80,77,80,71,73,80	76.9167	3.2551	0.4634
C1	79,80,77,80,82,70,74,78,79,75,78,80	77.6667	3.0637	1.184
C2	77,84,78,77,78,79,80,86,76,86,83,80	80.3333	3.6903	1.0062
C3	87,84,83,84,82,78,81,86,85,81,83,81	82.9167	2.4196	0.6409
C4	76,82,71,76,71,70,70,80,75,71,75,89	75.5	5.3266	2.2341
C5	90,92,95,91,83,87,90,92,85,87,89,96	89.75	3.7077	1.0014
C6	92,86,92,92,93,88,92,89,93,89,96,95	91.4167	2.8548	0.5452
C7	85,93,86,85,89,85,84,84,84,80,93,90	86.5	3.9688	0.454

#### 3.2.3. Calculation of the comprehensive cloud.

After obtaining the indicator weights and the digital characteristics of the assessment clouds, the digital characteristic values of the project’s comprehensive cloud were calculated, where Ex was 78.6933, En was 4.4848, and He was 1.2983. Using MATLAB, the cloud map characteristics of the standard clouds and the comprehensive cloud were plotted in the same coordinate system to determine the project’s fire safety grade, as shown in Fig 5. In this study, the number of cloud drops was set to 4,000, the comprehensive cloud was mainly concentrated within the interval (70,90]. Based on the average similarity values obtained from 10 independent repeated cloud similarity calculations (Ⅴ: 0, Ⅳ: 0, Ⅲ: 0.0045, Ⅱ: 0.5906, Ⅰ: 0.0029; the results of each calculation are provided in Appendix Table A11), the fire risk grade of the project was determined to be Grade II. The sensitivity analysis of He is presented in Appendix Table A12.

**Fig 5:**
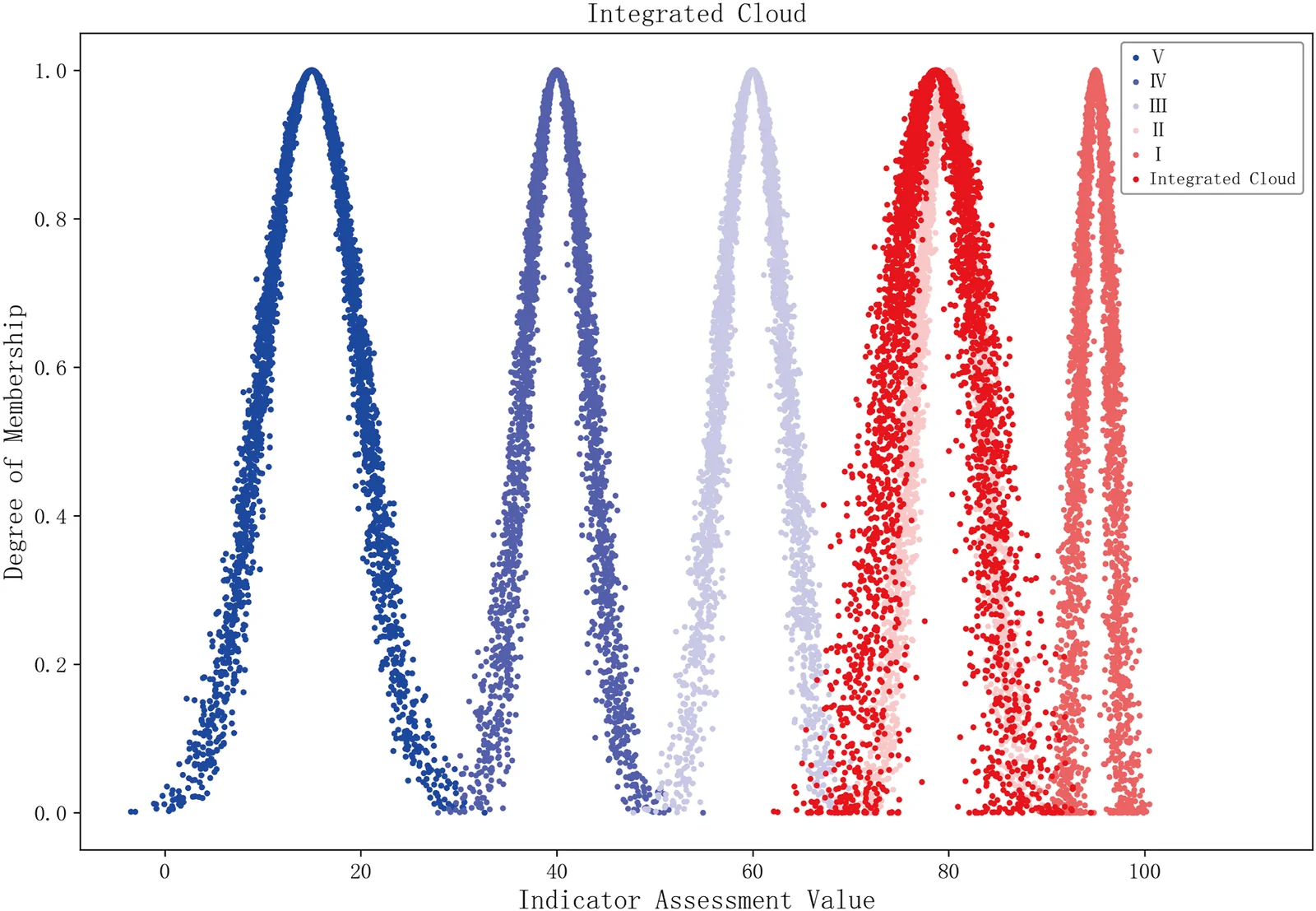
Integrated cloud and standard clouds.

### 3.3. Analysis of the assessment results

The assessment results indicate that the fire risk grade of the case-study building is Grade II, suggesting that its overall fire risk is relatively low and within an acceptable range. According to the final combined weights (Fig 6), hazard has the greatest influence on the overall fire risk, followed by vulnerability, emergency response and recovery capacity, and exposure. This indicates that the fire risk of the case-study high-rise building is mainly affected by fire-inducing factors and building vulnerability, while emergency response and recovery capacity also plays an important role in controlling fire consequences.

**Fig 6:**
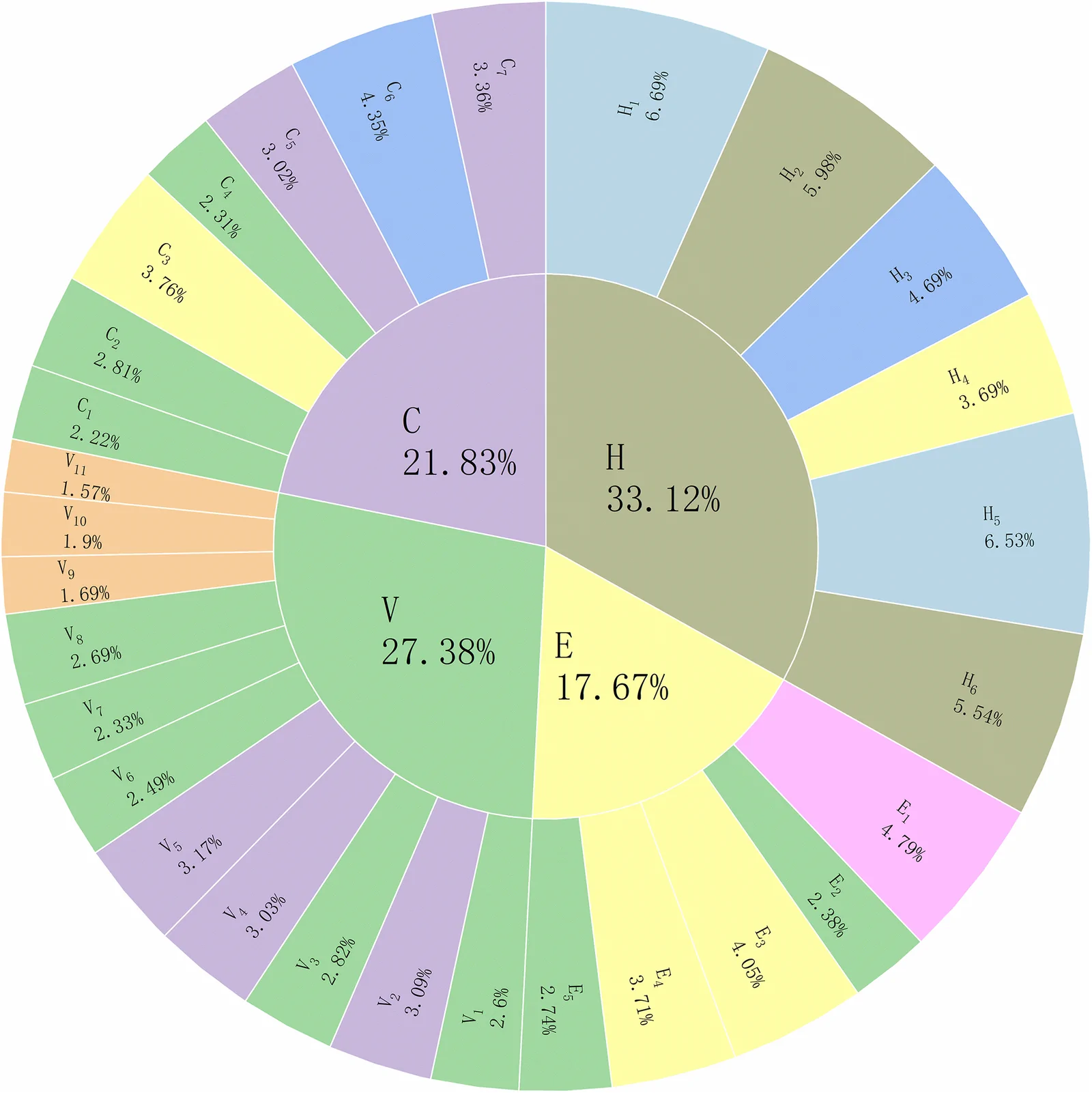
Indicator weight comparison.

Overall, the Grade II assessment result shows that the case-study building has a relatively low fire risk. However, risk-control measures should not focus only on a single factor. Instead, fire safety management should comprehensively address ignition-source control, fire-load reduction, façade fire risk control, evacuation capacity improvement, maintenance of fire-protection facilities, and coordination with external fire and rescue resources. These measures can further reduce the probability of fire occurrence, limit fire spread, improve evacuation efficiency, and enhance the overall fire safety level of high-rise buildings.

Although the dimension-level weighting results provide an overall understanding of the contribution of different risk dimensions, the indicator-level assessment results further reveal the specific weaknesses of the case-study building. According to the expert evaluation results, V7, V1, and E1 obtained relatively low expected values, with values of 67.50, 69.25, and 69.83, respectively. These results indicate that although the overall fire risk grade of the building was Grade II, several specific factors require further improvement. Specifically, the relatively low score of E1 reflects the influence of occupant exposure on evacuation demand and emergency response pressure. The low score of V1 indicates that the relatively high proportion of elderly residents and children increases evacuation difficulty due to their limited mobility and self-rescue capability. In addition, the low score of V7 suggests that the refuge-related safety capacity of the building, including the availability and effectiveness of auxiliary evacuation facilities, requires further improvement.

Based on the identified weak indicators, targeted risk mitigation measures should be implemented. For E1, evacuation organization and occupant management should be strengthened through measures such as optimizing evacuation planning, improving evacuation guidance, and conducting regular emergency drills to reduce evacuation pressure during fire events. For V1, specific assistance mechanisms for vulnerable occupants should be established, including prioritized evacuation arrangements, enhanced fire safety education, and improved emergency support during evacuation. For V7, attention should be given to the configuration and maintenance of refuge-related and auxiliary evacuation facilities, such as escape ladders, descent devices, emergency escape devices, escape ropes, and self-rescue respiratory protection equipment, according to the characteristics of the building.

Compared with previous fire risk assessment frameworks for large commercial buildings and shopping malls, the proposed indicator system places greater emphasis on the specific evacuation and rescue characteristics of high-rise buildings. In addition to conventional factors related to fire hazards, protection systems, and management, this framework incorporates vertical evacuation-related indicators and city-level external rescue capacity. These additions better reflect the long vertical evacuation distances and greater dependence on professional rescue forces in high-rise building fires.

## 4. Model comparison

Although the proposed method for assessing fire risk in high-rise buildings has been fully developed, further validation is still needed to confirm whether it can accurately reflect actual risk levels. To this end, we applied a mature fire risk index method (FRI) to assess the fire risk of the case-study building and compare the results obtained from the two methods [28]. In the FRI method, a higher risk score indicates better fire safety performance and a lower fire risk level. The fire risk classification criteria of the FRI method are presented in Table 7.

**Table 7:** Building fire risk levels in the FRI method.

Fire risk score R	1 ≤ R ≤ 1.5	1.5 < R ≤ 2.5	2.5 < R ≤ 3.5	3.5 < R ≤ 4.5	4.5 < R ≤ 5
Fire risk level	Ⅰ	Ⅱ	Ⅲ	Ⅳ	Ⅴ

The FRI method mainly constructs its indicator system from two aspects: disaster-inducing factors and loss-control factors. The loss-control factors are further divided into passive measures, active measures, fire management, and firefighting capability. This indicator structure has a certain correspondence with the dimensions of Integrated Disaster Risk Theory. Specifically, disaster-inducing factors mainly correspond to the hazard dimension in the proposed framework; passive measures and active measures mainly correspond to building vulnerability; and fire management and firefighting capability mainly correspond to emergency response and recovery capacity. In contrast, the FRI method does not establish an independent exposure dimension, and its reflection of exposure factors such as occupants, property, and evacuation demand is relatively indirect.

Following the selected fire-risk-index-based method, an on-site investigation of the case-study building was conducted. In combination with the as-built drawings and field investigation results, the scores of all indicators specified by this method were determined. The FRI method draws on international experience and has been adjusted according to local conditions. It is a fire-risk-index-based assessment method that is widely used to determine the fire risk level of buildings and to support property insurance underwriting and premium adjustment, thereby allowing insurance rates to reflect the actual risk status of insured buildings. Table 8 summarizes the assessment results obtained using the FRI method.

**Table 8:** Fire risk in high-rise buildings based on the fire risk index method: a case study.

Serial Number	Index	Score *S*_*i*_	Weight *ω_i_*	Index Score *R_i_* = *ω_i_* * *S*_*i*_
1	Gas Usage Mode	5	0.076	0.38
2	Personnel Competency	4	0.081	0.324
3	Electrical Equipment	4	0.095	0.38
4	Interior Decoration	4	0.039	0.156
5	Internal and External Insulation	3	0.04	0.12
6	Building Height	2	0.015	0.03
7	Building Age	5	0.033	0.165
8	External Environment	5	0.014	0.07
9	Fire Resistance Rating	4	0.07	0.28
10	Fire Compartmentation	5	0.03	0.15
11	Safe Evacuation	3	0.039	0.117
12	Fire Separation Distance	5	0.008	0.04
13	Safety Monitoring	3	0.077	0.231
14	Indoor Fire Hydrant Water Supply System	3	0.084	0.252
15	Portable Fire Extinguishers	2	0.093	0.186
16	Property Fire Safety Management	4	0.086	0.344
17	Fire Vehicle Access	3	0.038	0.114
18	Fire Brigade Combat Capability	2	0.044	0.088
19	Outdoor Fire Water Supply System	4	0.038	0.152
Assessment result	$\text{R}=\sum_{\text{i}=\text{1}}^{\text{n}}\omega_{i}*s_{i}=\text{3}.\text{5}\text{7}\text{9}$			

The proposed method produced an expectation value of the comprehensive cloud of 78.6933, and the maximum similarity corresponded to Grade II, indicating that the fire risk level of the case-study building was relatively low. The FRI method produced a fire risk value of 3.579, which was also classified as low fire risk according to the grading criteria of that method. For further numerical comparison, the FRI score was linearly normalized to a 0–100 scale, yielding a normalized value of 71.58. The absolute difference between this value and the expectation value of the comprehensive cloud obtained using the proposed method was 7.1133, corresponding to a relative deviation of approximately 9.04%. This numerical difference mainly arises from differences in the indicator structure, weighting method, and risk-grade determination procedure between the two methods. It should be noted that the comparison with the FRI method does not represent an independent validation of the proposed model. Both approaches involve expert-based judgments and were applied to the same case-study building. In addition, some assessment information and indicators are partially overlapping between the two methods. Therefore, the agreement between the two methods mainly reflects the consistency and plausibility of the proposed assessment framework.

Although the two methods differ in model structure and calculation procedure, they reached a consistent qualitative conclusion for the same case-study building, both indicating that its fire risk level was acceptable and relatively low. This consistency supports the feasibility and rationality of the proposed assessment method for high-rise building fire risk analysis based on the Integrated Disaster Risk Theory.

## 5. Conclusions

This study introduced Integrated Disaster Risk Theory into high-rise building fire risk assessment and constructed a fire risk assessment indicator system specifically applicable to high-rise buildings. The proposed indicator system was developed from four dimensions: hazard, exposure, vulnerability, and emergency response and recovery capacity. It reflects the specific characteristics of high-rise building fires.

Compared with ordinary buildings, high-rise buildings face greater difficulties in evacuation, fire suppression, and rescue operations once a fire occurs. Therefore, this study gives more comprehensive consideration to the emergency response and recovery capacity dimension. In addition to building-level internal fire safety management and initial response capacity, city-level external fire and rescue capacity was incorporated into the indicator system. The compatibility between firefighting and rescue equipment and building characteristics was also included to reflect the adaptability of professional rescue resources to high-rise building fire scenarios.

The proposed indicator system and assessment procedure were applied to a typical high-rise residential building in Changchun City. The comprehensive cloud digital characteristics were Ex 78.6933, En 4.4848, and He 1.2983. The maximum similarity corresponded to Grade II, indicating a relatively low fire risk. This result was consistent with that obtained using an established fire risk index-based method, supporting the plausibility and consistency of the proposed assessment model.

The main contribution of this study lies in refining the fire risk assessment indicator system according to the scenario-specific characteristics of high-rise buildings and strengthening the consideration of both internal and external emergency response and recovery capacities. However, the empirical analysis was based on a single high-rise residential building, which limits the generalizability of the present findings. Future research should further validate the proposed indicator system using buildings with different functions, heights, regions, and management conditions, and may incorporate real-time monitoring data, building information modeling, Internet of Things sensing data, and fire incident databases to support more dynamic fire risk assessment.

## Supporting information

S1 File Appendix Table 1-12. (DOCX)
