# Supplementary material for: Fire risk assessment for high-rise buildings based on Integrated Disaster Risk Theory
Source: PLoS One. 2026 Oct 5;21(10):e0358941. doi: 10.1371/journal.pone.0358941 (PMC13637918; doi:10.1371/journal.pone.0358941)
Supplement: Supplementary file 1 [file pone.0358941.s001.docx]

**Appendix Table A1. Weights of primary indicators calculated using FAHP.**

|  | **H** | **E** | **V** | **C** | $\text{I(A,}\text{W}^{\text{*}}\text{)}$ |
| --- | --- | --- | --- | --- | --- |
| **H** | 0.5 | 0.8 | 0.6 | 0.7 | $\text{I(A,}\text{W}^{\text{*}}\text{)}$=0.0831≤0.1 |
| **E** | 0.2 | 0.5 | 0.3 | 0.4 |  |
| **V** | 0.4 | 0.7 | 0.5 | 0.6 |  |
| **C** | 0.3 | 0.6 | 0.4 | 0.5 |  |
| **-** |  |  |  |  |  |
| **ω** | 0.3 | 0.2 | 0.2667 | 0.2333 |  |

**Appendix Table A2. Weights of hazard indicators calculated using FAHP.**

|  | **H_1_** | **H_2_** | **H_3_** | **H_4_** | **H_5_** | **H_6_** | $\text{I(A,}\text{W}^{\text{*}}\text{)}$ |
| --- | --- | --- | --- | --- | --- | --- | --- |
| **H_1_** | 0.5 | 0.6 | 0.7 | 0.8 | 0.5 | 0.6 | $\text{I(A,}\text{W}^{\text{*}}\text{)}$=0.0756≤0.1 |
| **H_2_** | 0.4 | 0.5 | 0.6 | 0.7 | 0.5 | 0.5 |  |
| **H_3_** | 0.3 | 0.4 | 0.5 | 0.6 | 0.3 | 0.4 |  |
| **H_4_** | 0.2 | 0.3 | 0.4 | 0.5 | 0.3 | 0.3 |  |
| **H_5_** | 0.5 | 0.5 | 0.7 | 0.7 | 0.5 | 0.6 |  |
| **H_6_** | 0.4 | 0.5 | 0.6 | 0.7 | 0.4 | 0.5 |  |
| **ω** | 0.19 | 0.1733 | 0.15 | 0.1333 | 0.1833 | 0.17 |  |

**Appendix Table A3. Weights of exposure indicators calculated using FAHP.**

|  | **E_1_** | **E_2_** | **E_3_** | **E_4_** | **E_5_** | $\text{I(A,}\text{W}^{\text{*}}\text{)}$ |
| --- | --- | --- | --- | --- | --- | --- |
| **E_1_** | 0.5 | 0.8 | 0.6 | 0.6 | 0.8 | $\text{I(A,}\text{W}^{\text{*}}\text{)}$=0.083≤0.1 |
| **E_2_** | 0.2 | 0.5 | 0.3 | 0.3 | 0.5 |  |
| **E_3_** | 0.4 | 0.7 | 0.5 | 0.5 | 0.7 |  |
| **E_4_** | 0.4 | 0.7 | 0.5 | 0.5 | 0.6 |  |
| **E_5_** | 0.2 | 0.5 | 0.3 | 0.4 | 0.5 |  |
| **ω** | 0.24 | 0.165 | 0.215 | 0.21 | 0.17 |  |

**Appendix Table A4. Weights of vulnerability indicators calculated using FAHP.**

|  | **V_1_** | **V_2_** | **V_3_** | **V_4_** | **V_5_** | **V_6_** | **V_7_** | **V_8_** | **V_9_** | **V_10_** | **V_11_** | $\text{I(A,}\text{W}^{\text{*}}\text{)}$ |
| --- | --- | --- | --- | --- | --- | --- | --- | --- | --- | --- | --- | --- |
| **V_1_** | 0.5 | 0.4 | 0.5 | 0.5 | 0.4 | 0.5 | 0.5 | 0.5 | 0.6 | 0.6 | 0.7 | $\text{I(A,}\text{W}^{\text{*}}\text{)}$=0.062≤0.1 |
| **V_2_** | 0.6 | 0.5 | 0.5 | 0.5 | 0.5 | 0.6 | 0.6 | 0.5 | 0.7 | 0.7 | 0.7 |  |
| **V_3_** | 0.5 | 0.5 | 0.5 | 0.5 | 0.5 | 0.5 | 0.6 | 0.5 | 0.7 | 0.6 | 0.7 |  |
| **V_4_** | 0.5 | 0.5 | 0.5 | 0.5 | 0.5 | 0.6 | 0.6 | 0.5 | 0.7 | 0.6 | 0.7 |  |
| **V_5_** | 0.6 | 0.5 | 0.5 | 0.5 | 0.5 | 0.6 | 0.6 | 0.6 | 0.7 | 0.7 | 0.7 |  |
| **V_6_** | 0.5 | 0.4 | 0.5 | 0.4 | 0.4 | 0.5 | 0.5 | 0.5 | 0.6 | 0.6 | 0.7 |  |
| **V_7_** | 0.5 | 0.4 | 0.4 | 0.4 | 0.4 | 0.5 | 0.5 | 0.5 | 0.6 | 0.6 | 0.6 |  |
| **V_8_** | 0.5 | 0.5 | 0.5 | 0.5 | 0.4 | 0.5 | 0.5 | 0.5 | 0.7 | 0.6 | 0.7 |  |
| **V_9_** | 0.4 | 0.3 | 0.3 | 0.3 | 0.3 | 0.4 | 0.4 | 0.3 | 0.5 | 0.5 | 0.5 |  |
| **V_10_** | 0.4 | 0.3 | 0.4 | 0.4 | 0.3 | 0.4 | 0.4 | 0.4 | 0.5 | 0.5 | 0.6 |  |
| **V_11_** | 0.3 | 0.3 | 0.3 | 0.3 | 0.3 | 0.3 | 0.4 | 0.3 | 0.5 | 0.4 | 0.5 |  |
| **ω** | 0.0927 | 0.0991 | 0.0964 | 0.0973 | 0.1 | 0.0918 | 0.09 | 0.0945 | 0.0791 | 0.0827 | 0.0764 |  |

**Appendix Table A5. Weights of emergency response and recovery capacity indicators calculated using FAHP.**

|  | **C_1_** | **C_2_** | **C_3_** | **C_4_** | **C_5_** | **C_6_** | **C_7_** | $\text{I(A,}\text{W}^{\text{*}}\text{)}$ |
| --- | --- | --- | --- | --- | --- | --- | --- | --- |
| **C_1_** | 0.5 | 0.4 | 0.3 | 0.5 | 0.4 | 0.2 | 0.3 | $\text{I(A,}\text{W}^{\text{*}}\text{)}$=0.0799≤0.1 |
| **C_2_** | 0.6 | 0.5 | 0.4 | 0.6 | 0.5 | 0.3 | 0.4 |  |
| **C_3_** | 0.7 | 0.6 | 0.5 | 0.7 | 0.6 | 0.4 | 0.6 |  |
| **C_4_** | 0.5 | 0.4 | 0.3 | 0.5 | 0.4 | 0.2 | 0.4 |  |
| **C_5_** | 0.6 | 0.5 | 0.4 | 0.6 | 0.5 | 0.3 | 0.5 |  |
| **C_6_** | 0.8 | 0.7 | 0.6 | 0.8 | 0.7 | 0.5 | 0.6 |  |
| **C_7_** | 0.7 | 0.6 | 0.4 | 0.6 | 0.5 | 0.4 | 0.5 |  |
| **ω** | 0.1214 | 0.1381 | 0.1571 | 0.1238 | 0.1405 | 0.1714 | 0.1476 |  |

**Appendix Table A6. Weights of primary indicators calculated using SEWM.**

|  | **H** | **E** | **V** | **C** |
| --- | --- | --- | --- | --- |
| Group 1 | 1 | 4 | 2 | 3 |
| Group 2 | 1 | 2 | 3 | 4 |
| Group 3 | 2 | 4 | 1 | 3 |
| Group 4 | 1 | 4 | 2 | 3 |
| Average cognition degree $\text{b}_{\text{j}}$ | 0.9653 | 0.5383 | 0.8513 | 0.6196 |
| Maximum | 1.0000 | 0.8614 | 1 | 0.6826 |
| Maximum-$\text{b}_{\text{j}}$ | 0.0347 | 0.323 | 0.1487 | 0.063 |
| Minimum | 0.8614 | 0.4307 | 0.6826 | 0.4307 |
| $\text{b}_{\text{j}}$-Minimum | 0.1040 | 0.1077 | 0.1687 | 0.1889 |
| $\text{Q}_{\text{j}}$ | 0.0693 | 0.2153 | 0.1587 | 0.126 |
| 1-$\text{Q}_{\text{j}}$ | 0.9307 | 0.7847 | 0.8413 | 0.874 |
| $\text{k}_{\text{j}}$ | 0.8984 | 0.4224 | 0.7162 | 0.5416 |
| Weight | 0.3484 | 0.1638 | 0.2778 | 0.21 |

**Appendix Table A7. Weights of hazard indicators calculated using SEWM.**

|  | **H_1_** | **H_2_** | **H_3_** | **H_4_** | **H_5_** | **H_6_** |
| --- | --- | --- | --- | --- | --- | --- |
| Group 1 | 1 | 2 | 4 | 5 | 1 | 3 |
| Group 2 | 1 | 3 | 5 | 6 | 2 | 4 |
| Group 3 | 2 | 3 | 4 | 5 | 1 | 3 |
| Group 4 | 1 | 2 | 4 | 5 | 2 | 3 |
| Average cognition degree $\text{b}_{\text{j}}$ | 0.9802 | 0.8739 | 0.6755 | 0.5125 | 0.9604 | 0.7984 |
| Maximum | 1.0000 | 0.9208 | 0.7124 | 0.5646 | 1.0000 | 0.8271 |
| Maximum-$\text{b}_{\text{j}}$ | 0.0198 | 0.0468 | 0.0370 | 0.0521 | 0.0396 | 0.0287 |
| Minimum | 0.9208 | 0.8271 | 0.5646 | 0.3562 | 0.9208 | 0.7124 |
| $\text{b}_{\text{j}}$-Minimum | 0.0594 | 0.0468 | 0.1109 | 0.1563 | 0.0396 | 0.0860 |
| $\text{Q}_{\text{j}}$ | 0.0396 | 0.0468 | 0.0739 | 0.1042 | 0.0396 | 0.0573 |
| 1-$\text{Q}_{\text{j}}$ | 0.9604 | 0.9532 | 0.9261 | 0.8958 | 0.9604 | 0.9427 |
| $\text{k}_{\text{j}}$ | 0.9414 | 0.8330 | 0.6255 | 0.4591 | 0.9224 | 0.7526 |
| Weight | 0.2076 | 0.1837 | 0.1380 | 0.1013 | 0.2034 | 0.1660 |

**Appendix Table A8. Weights of exposure indicators calculated using SEWM.**

|  | **E_1_** | **E_2_** | **E_3_** | **E_4_** | **E_5_** |
| --- | --- | --- | --- | --- | --- |
| Group 1 | 1 | 4 | 3 | 2 | 5 |
| Group 2 | 1 | 5 | 2 | 4 | 3 |
| Group 3 | 1 | 5 | 3 | 2 | 4 |
| Group 4 | 2 | 5 | 1 | 3 | 4 |
| Average cognition degree $\text{b}_{\text{j}}$ | 0.9746 | 0.4434 | 0.8614 | 0.7958 | 0.5967 |
| Maximum | 1.0000 | 0.6131 | 1.0000 | 0.8982 | 0.7737 |
| Maximum-$\text{b}_{\text{j}}$ | 0.0254 | 0.1697 | 0.1386 | 0.1024 | 0.1770 |
| Minimum | 0.8982 | 0.3869 | 0.7737 | 0.6131 | 0.3869 |
| $\text{b}_{\text{j}}$-Minimum | 0.0763 | 0.0566 | 0.0877 | 0.1827 | 0.2099 |
| $\text{Q}_{\text{j}}$ | 0.0509 | 0.1131 | 0.1131 | 0.1425 | 0.1934 |
| 1-$\text{Q}_{\text{j}}$ | 0.9491 | 0.8869 | 0.8869 | 0.8575 | 0.8066 |
| $\text{k}_{\text{j}}$ | 0.9250 | 0.3933 | 0.7639 | 0.6824 | 0.4813 |
| Weight | 0.2850 | 0.1212 | 0.2354 | 0.2102 | 0.1483 |

**Appendix Table A9. Weights of vulnerability indicators calculated using SEWM.**

|  | **V_1_** | **V_2_** | **V_3_** | **V_4_** | **V_5_** | **V_6_** | **V_7_** | **V_8_** | **V_9_** | **V_10_** | **V_11_** |
| --- | --- | --- | --- | --- | --- | --- | --- | --- | --- | --- | --- |
| Group 1 | 5 | 2 | 4 | 3 | 1 | 7 | 6 | 4 | 8 | 8 | 9 |
| Group 2 | 3 | 1 | 2 | 2 | 1 | 4 | 4 | 2 | 6 | 5 | 5 |
| Group 3 | 4 | 1 | 5 | 3 | 2 | 7 | 8 | 6 | 11 | 9 | 10 |
| Group 4 | 7 | 3 | 4 | 1 | 2 | 6 | 8 | 5 | 9 | 10 | 11 |
| Average cognition degree $\text{b}_{\text{j}}$ | 0.8422 | 0.9729 | 0.8926 | 0.9546 | 0.9825 | 0.7774 | 0.7407 | 0.8673 | 0.5669 | 0.6211 | 0.5289 |
| Maximum | 0.9266 | 1.0000 | 0.9650 | 1.0000 | 1.0000 | 0.8842 | 0.8842 | 0.9650 | 0.7831 | 0.8368 | 0.8368 |
| Maximum-$\text{b}_{\text{j}}$ | 0.0844 | 0.0271 | 0.0724 | 0.0454 | 0.0175 | 0.1069 | 0.1436 | 0.0977 | 0.2162 | 0.2157 | 0.3079 |
| Minimum | 0.7211 | 0.9266 | 0.8368 | 0.9266 | 0.9650 | 0.7211 | 0.6477 | 0.7831 | 0.2789 | 0.4421 | 0.2789 |
| $\text{b}_{\text{j}}$-Minimum | 0.1211 | 0.0463 | 0.0557 | 0.0279 | 0.0175 | 0.0563 | 0.0930 | 0.0842 | 0.2880 | 0.1790 | 0.2500 |
| $\text{Q}_{\text{j}}$ | 0.1028 | 0.0367 | 0.0641 | 0.0367 | 0.0175 | 0.0816 | 0.1183 | 0.0909 | 0.2521 | 0.1974 | 0.2789 |
| 1-$\text{Q}_{\text{j}}$ | 0.8972 | 0.9633 | 0.9359 | 0.9633 | 0.9825 | 0.9184 | 0.8817 | 0.9091 | 0.7479 | 0.8026 | 0.7211 |
| $\text{k}_{\text{j}}$ | 0.7556 | 0.9372 | 0.8354 | 0.9195 | 0.9653 | 0.7139 | 0.6531 | 0.7884 | 0.4240 | 0.4985 | 0.3814 |
| Weight | 0.0960 | 0.1191 | 0.1061 | 0.1168 | 0.1226 | 0.0907 | 0.0830 | 0.1001 | 0.0539 | 0.0633 | 0.0484 |

**Appendix Table A10. Weights of emergency response and recovery capacity indicators calculated using SEWM.**

|  | **C_1_** | **C_2_** | **C_3_** | **C_4_** | **C_5_** | **C_6_** | **C_7_** |
| --- | --- | --- | --- | --- | --- | --- | --- |
| Group 1 | 7 | 3 | 4 | 6 | 2 | 1 | 5 |
| Group 2 | 5 | 6 | 2 | 7 | 3 | 1 | 4 |
| Group 3 | 7 | 4 | 3 | 5 | 6 | 2 | 1 |
| Group 4 | 5 | 6 | 2 | 3 | 4 | 1 | 2 |
| Average cognition degree $\text{b}_{\text{j}}$ | 0.5000 | 0.6731 | 0.8768 | 0.5975 | 0.7749 | 0.9839 | 0.8441 |
| Maximum | 0.6667 | 0.8617 | 0.9358 | 0.8617 | 0.9358 | 1.0000 | 1.0000 |
| Maximum-$\text{b}_{\text{j}}$ | 0.1667 | 0.1886 | 0.0590 | 0.2642 | 0.1609 | 0.0161 | 0.1559 |
| Minimum | 0.3333 | 0.5283 | 0.7740 | 0.3333 | 0.5283 | 0.9358 | 0.6667 |
| $\text{b}_{\text{j}}$-Minimum | 0.1667 | 0.1447 | 0.1028 | 0.2642 | 0.2466 | 0.0482 | 0.1774 |
| $\text{Q}_{\text{j}}$ | 0.1667 | 0.1667 | 0.0809 | 0.2642 | 0.2037 | 0.0321 | 0.1667 |
| 1-$\text{Q}_{\text{j}}$ | 0.8333 | 0.8333 | 0.9191 | 0.7358 | 0.7963 | 0.9679 | 0.8333 |
| $\text{k}_{\text{j}}$ | 0.4167 | 0.5609 | 0.8059 | 0.4397 | 0.6171 | 0.9524 | 0.7034 |
| Weight | 0.0927 | 0.1248 | 0.1792 | 0.0978 | 0.1372 | 0.2118 | 0.1565 |

**Appendix Table A11. Results of 10 independent repeated cloud similarity calculations.**

|  | Ⅰ | Ⅱ | Ⅲ | Ⅳ | Ⅴ |
| --- | --- | --- | --- | --- | --- |
| 1 | 0.0039 | 0.5831 | 0.0045 | 0 | 0 |
| 2 | 0.0027 | 0.5944 | 0.006 | 0 | 0 |
| 3 | 0.0023 | 0.5922 | 0.0052 | 0 | 0 |
| 4 | 0.0023 | 0.5818 | 0.0046 | 0 | 0 |
| 5 | 0.0024 | 0.598 | 0.0042 | 0 | 0 |
| 6 | 0.004 | 0.5947 | 0.0037 | 0 | 0 |
| 7 | 0.0028 | 0.5904 | 0.0031 | 0 | 0 |
| 8 | 0.0029 | 0.5865 | 0.0046 | 0 | 0 |
| 9 | 0.0028 | 0.5997 | 0.0046 | 0 | 0 |
| 10 | 0.0033 | 0.5855 | 0.0042 | 0 | 0 |
| Average value | 0.0029 | 0.5906 | 0.0045 | 0 | 0 |
| Standard deviation | 0.0006 | 0.0062 | 0.0008 | 0 | 0 |

**Appendix Table A12. Sensitivity analysis of similarity results under different He values.**

| He | Ⅰ | Ⅱ | Ⅲ | Ⅳ | Ⅴ |
| --- | --- | --- | --- | --- | --- |
| 0.1 | 0.0045 | 0.5839 | 0.0053 | 0 | 0 |
| 0.2 | 0.0025 | 0.5979 | 0.006 | 0 | 0 |
| 0.3 | 0.004 | 0.593 | 0.0054 | 0 | 0 |
| 0.4 | 0.0045 | 0.5863 | 0.0047 | 0 | 0 |
| 0.5 | 0.0027 | 0.5944 | 0.006 | 0 | 0 |
| 0.6 | 0.0023 | 0.5818 | 0.0046 | 0 | 0 |
| 0.7 | 0.0037 | 0.5892 | 0.0048 | 0 | 0 |
| 0.8 | 0.0029 | 0.5865 | 0.0046 | 0 | 0 |
| 0.9 | 0.0023 | 0.5818 | 0.0046 | 0 | 0 |
| 1 | 0.0027 | 0.5944 | 0.006 | 0 | 0 |
