# Supplementary material for: Fire risk assessment for high-rise buildings based on Integrated Disaster Risk Theory
Source: PLoS One. 2026 Oct 5;21(10):e0358941. doi: 10.1371/journal.pone.0358941 (PMC13637918; doi:10.1371/journal.pone.0358941)
Supplement: Supplementary file 2 [file pone.0358941.s003.docx]

**Original Manuscript Number:** PONE-D-26-13643

**Original Article Title: “Fire risk assessment for high-rise buildings based on Integrated Disaster Risk Theory**”

Dear Editor,

We would like to express our sincere gratitude to you and the reviewers for the valuable time and constructive comments devoted to our manuscript (Manuscript Number: PONE-D-26-13643). The insightful suggestions have been instrumental in helping us significantly improve the quality of our work.

We have carefully considered all the comments and have revised the manuscript accordingly. Our point-by-point responses to each comment are detailed below.

We believe the manuscript is now strengthened and hope it meets the journal's standards for publication.

Thank you for your consideration.

Best regards,

Zonglai Liu et al.

**Reviewer#1, Concern # 1: The innovation points are vaguely stated, and the incremental contribution compared to existing methods is not clearly explained.**

**Author response:** Thank you for your constructive feedback. The main objective of this study is to construct a fire risk assessment indicator system specifically applicable to high-rise buildings. Compared with ordinary buildings, high-rise buildings face greater challenges in evacuation, fire suppression, and rescue operations after a fire occurs. Therefore, the emergency response and recovery capacity of high-rise buildings requires more comprehensive consideration. In the revised manuscript, this dimension is strengthened by incorporating both building-level internal fire safety capacity and city-level external fire and rescue capacity. Moreover, the compatibility between firefighting equipment and building characteristics is included to reflect the adaptability of professional rescue resources to high-rise building fire scenarios.

**Author action:** We have revised the abstract (lines 21-23, 29-31), introduction (lines 87-116), and conclusion (lines 525-547) in the main text.

**Reviewer#1, Concern # 2:** **Related research should be more comprehensive, such as Potential of large language models in blockchain-based supply chain finance[J]. Enterprise Information Systems, 2025: 2541199. and FIRE RISK ASSESSMENT FOR LARGE COMMERCI`AL COMPLEXES BASED ON INTEGRATED DISASTER RISK THEORY USING GAME-THEORETIC COMBINATION WEIGHTING AND THE CLOUD MODEL. International Journal of Infrastructure Research and Management, 2024, 12(1), 44-71.**

**Author response:** Thank you for your valuable suggestion. We have carefully reviewed and fully considered the relevant studies suggested by the reviewer. Among them, the study on fire risk assessment for large commercial complexes is highly relevant to the present work. However, the research object and the logic of indicator construction in our study are clearly different from those of that study. Previous research mainly focused on large commercial complexes, such as shopping malls and mixed-use commercial buildings, whose major fire risk characteristics include high occupant density, complex commercial functions, high fire load, and long internal travel distances. In contrast, the present study focuses specifically on fire risk assessment for high-rise buildings. High-rise buildings face more prominent challenges in vertical evacuation, firefighting at height, smoke spread through vertical shafts, external rescue accessibility, and dependence on professional fire and rescue forces. Therefore, the main contribution of this study is not the simple reuse of existing assessment methods, but the reconstruction of a more targeted fire risk assessment index system according to the specific fire risk characteristics of high-rise buildings. In the revised manuscript, we further adjusted and improved the indicator system so that it can better reflect the risk characteristics of high-rise building fire scenarios. These revisions help distinguish the present study from previous commercial-complex-oriented assessment frameworks and improve the applicability of the indicator system to high-rise building fire risk assessment.

**Author action:** We revised the index section of the paper to reflect the differences from previous studies (lines 154-252). The revised index are shown in Table 1:

**Table 1. High-rise Building Fire Risk Assessment Index System.**

| **HRFRI** | **Factor** | **Index** |
| --- | --- | --- |
| High-rise Building Fire Risk Assessment Index System | Hazard （H） | Occupant activity intensity （H_1_） |
|  |  | Electrical facilities（H_2_） |
|  |  | Gas facilities（H_3_） |
|  |  | External environment（H_4_） |
|  |  | Indoor fire load(H_5_) |
|  |  | Building thermal insulation and exterior wall decoration（H_6_） |
|  | Exposure（E） | Population size（E_1_） |
|  |  | Building functional complexity（E_2_） |
|  |  | Evacuation facility capacity（E_3_） |
|  |  | Tangible assets of the building(E_4_) |
|  |  | Intangible assets of the building (E_5_) |
|  | Vulnerability（V） | Proportion of elderly and children（V_1_） |
|  |  | Building height（V_2_） |
|  |  | Building service life（V_3_） |
|  |  | Fire compartment（V_4_） |
|  |  | Fire-resistance rating（V_5_） |
|  |  | Evacuation routes and evacuation guidance facilities（V_6_） |
|  |  | Refuge floors and refuge facilities（V_7_） |
|  |  | Water-supply facilities（V_8_） |
|  |  | Automatic fire alarm system（V_9_） |
|  |  | Automatic sprinkler system（V_10_） |
|  |  | Ventilation and smoke exhaust system（V_11_） |
|  | Emergency response and recovery capacity（C） | Fire management system（C_1_） |
|  |  | Fire-safety inspection（C_2_） |
|  |  | Fire-facility maintenance（C_3_） |
|  |  | Construction status of micro fire stations（C_4_） |
|  |  | Comprehensive rescue capacity of the local fire station（C_5_） |
|  |  | Firefighting response time（C_6_） |
|  |  | Compatibility between firefighting equipment and building characteristics （C_7_） |

**Reviewer#1, Concern # 3:** **The exposure dimension only has 3 indicators, which lack sufficient coverage and lacks key items such as economic losses and evacuation channels.**

**Author response:** Thank you for your constructive comment. We agree that the exposure dimension in the original manuscript was not sufficiently comprehensive and did not fully cover the disaster-bearing bodies that may be directly affected after a high-rise building fire occurs. In disaster risk research, exposure mainly refers to the people and property at risk that are located within the potential impact range of a hazard. Therefore, in the revised manuscript, we expanded and refined the exposure dimension according to the characteristics of high-rise building fire scenarios. The revised exposure dimension includes five indicators from four aspects: human exposure, functional exposure, evacuation exposure, and property exposure. Population size within the building (E_1_) is used to reflect the number of occupants who may be threatened in the event of a fire. Building functional complexity (E_2_) is used to reflect the coupling of fire risk among different functional spaces. The newly added evacuation facility capacity (E_3_) is used to reflect the ability of stairwells, evacuation corridors, safety exits, and other evacuation facilities to meet occupant evacuation demand. In addition, tangible assets of the building (E_4_) and intangible assets of the building (E_5_) were added to characterize potential direct and indirect economic losses, respectively.

**Author action:** We have revised the exposure-related assessment index and provided an explanation of them (lines 191-211).

**Reviewer#1, Concern # 4:** **The FAHP consistency test results are not given, and the validity of the expert judgment matrix cannot be judged.**

**Author response:** Thank you for your constructive comment. In the revised manuscript, we have supplemented the consistency test results of the FAHP judgment matrices.

**Author action:** We have added all the results obtained using FAHP to Appendix Tables 1-5 of the supporting information.

**Reviewer#1, Concern # 5:** **The cloud similarity calculation does not specify the number of repetitions and stability verification, and the results are not credible enough.**

**Author response:** Thank you for your valuable suggestion. We agree that the original manuscript did not provide sufficient details on the cloud similarity calculation process and its results, which may have affected the reliability of the assessment results. Therefore, in the revised manuscript, we supplemented the number of cloud drops used in the cloud similarity calculation. Specifically, 4,000 cloud drops were generated for each similarity calculation. In addition, we clearly reported the similarity results between the comprehensive cloud and the five standard-grade clouds, namely Grade V: 0, Grade IV: 0, Grade III: 0.0046, Grade II: 0.5898, and Grade I: 0.0039. The results show that the comprehensive cloud had the highest similarity with the Grade II standard cloud; therefore, the fire risk grade of the project was determined as Grade II.

**Author action:** In section 3.2.3 of the main text, we supplemented the calculated number of cloud droplets (4000) and the calculation results of cloud similarity (V:0, IV:0, III:0.0046, II:0.5898, I:0.0039) to enhance the reliability of the results.

**Reviewer#2, Concern # 1:** **The comprehensive disaster risk theory is directly copied to the fire of high-rise buildings. The adaptability of the theory and the formation mechanism of the fire has not been proved, and the dimensional division is mechanical and untargeted. It is recommended to supplement a paragraph to argue how the four dimensions of the comprehensive disaster risk theory can be adapted to man-made fires in high-rise buildings, and clarify the corresponding logic of "danger - fire triggers, exposure - personnel and property, vulnerability - building fire resistance, emergency response capacity - internal and external rescue".**

**Author response:** Thank you for your constructive comment. We agree that the original manuscript did not sufficiently explain the applicability of Integrated Disaster Risk Theory to high-rise building fires, which may have made the dimensional division appear somewhat mechanical. In the revised manuscript, we supplemented the relevant explanation to further clarify why high-rise building fires can be incorporated into the framework of Integrated Disaster Risk Theory and how the four dimensions correspond to the fire risk formation mechanism. We consider building fires to be both urban safety accidents and emergency events. From the perspective of disaster science, they can be regarded as a type of human-induced disaster. Therefore, this study understands high-rise building fire risk as the result of the combined effects of fire-inducing factors, disaster-bearing bodies, the vulnerability of disaster-bearing bodies, and emergency response and recovery capacity. Within this framework, hazard mainly corresponds to the inducing conditions for fire occurrence and development; exposure mainly corresponds to the people and property that may be affected by a fire; vulnerability mainly corresponds to the susceptibility of occupants and buildings to damage under fire conditions; and emergency response and recovery capacity corresponds to the internal and external capacity for disaster prevention, resistance, emergency response, and recovery.

**Author action:** We have provided a detailed description of the application of the theory in Section 2.1 of the revised manuscript (lines 154-252).

**Reviewer#2, Concern # 2:** **FAHP does not clearly show the basis for the calculation results of the compatibility index and the consistency test threshold α; the structural entropy weighting method does not disclose the expert grouping method and the original scoring data; the solution process of the linear equation system of the combination weight of the game theory and the coefficient normalisation steps are missing; the cloud model superentropy He fixed takes 0.5 Without any basis, the stability of cloud similarity calculation has not been verified. It is recommended to supplement the specific value of the compatibility index I (A,W*) of FAHP to explain the reason for the selection of α=0.1; clarify the setting basis of He=0.5 in the cloud model, and add 1-2 cloud similarity repeat tests to verify the stability; and complete the linear square of the weight of game theory. The result of solving the matrix and coefficient of the program group.**

**Author response:** Thank you for the reviewer’s careful reading and constructive comments. In response to these concerns, we have made corresponding supplements in the revised manuscript and the Supporting Information. First, for the FAHP method, we supplemented the fuzzy complementary judgment matrices and the specific values of the compatibility index I(A,W*) in the Supporting Information. The consistency-test threshold was set as α = 0.1 because this value is commonly adopted in the consistency test of fuzzy complementary judgment matrices, and a smaller α indicates a stricter consistency requirement. Second, for the structural entropy weight method, the 12 experts were divided into four groups according to their professional backgrounds, and the original ranking matrices were added to the Supporting Information. Third, for the cloud model, we specified the number of cloud drops used in the cloud similarity calculation and provided the similarity values between the comprehensive cloud and the five standard clouds. The hyper-entropy parameter He was set to 0.5 following the commonly used setting in cloud-model-based risk assessment studies. This parameter is used to characterize the uncertainty of entropy in the standard evaluation clouds. In addition, the similarity corresponding to Grade II was substantially higher than those of the other grades, indicating that the assessment result was not located near the boundary between adjacent risk grades and that the final grade determination was relatively stable under the adopted calculation setting. Finally, regarding the game-theoretic combination weighting method, we agree that the solution process of the linear equation system and the coefficient normalization should be clarified. To maintain the readability and conciseness of the manuscript, the detailed numerical solution process was not expanded in the main text. The linear equation system of the primary index: $\left[\begin{matrix} 0.2556 & 0.2604 \\ 0.2604 & 0.2695 \end{matrix}\right]\left[\begin{matrix} \text{α1} \\ \text{α2} \end{matrix}\right]=\left[\begin{matrix} 0.2556 \\ 0.2695 \end{matrix}\right]$, the normalized coefficients are 0.3571 and 0.6429, respectively, and the second-order index linear equation system $\left[\begin{matrix} 0.6092 & 0.6198 \\ 0.6198 & 0.6443 \end{matrix}\right]\left[\begin{matrix} \text{α3} \\ \text{α4} \end{matrix}\right]=\left[\begin{matrix} 0.6092 \\ 0.6443 \end{matrix}\right]$,the normalized coefficients are 0.3122 and 0.6878, respectively.

**Author action:** We have added specific calculations for FAHP and SEWM in the revised draft (Appendix Table 1-10 for Supporting information). In section 3.2.3 of the main text, we supplemented the calculated number of cloud droplets (4000) and the calculation results of cloud similarity (V:0, IV:0, III:0.0046, II:0.5898, I:0.0039) to enhance the reliability of the results.

**Reviewer#2, Concern # 3:** **Only one residential high-rise building in Changchun is used as a case, and there is no multi-building type and multi-regional verification; 12 experts did not disclose the professional background, scoring standards and scoring consistency; the comparison with the existing method is only at the result level, and there is no process alignment and statistical inspection. It is recommended to clarify the field (fire engineering / construction safety / emergency management) and years of practice of the 12 experts; supplement the details of the field survey of fire hazards of case construction, improve the original data table of expert scoring; add indicator alignment instructions and statistical analysis of result differences when comparing and verification.**

**Author response:** Thank you for this valuable comment. In the revised manuscript, we clarified the professional background and working experience of the 12 experts. All invited experts had more than three years of professional experience in fire safety, emergency management, or building fire-protection operations. The expert panel consisted of three university researchers specializing in safety-related fields, three frontline firefighters from fire and rescue departments, three professionals from third-party fire safety institutions, and three fire-facility operators from property management companies. In addition, we supplemented the original expert scoring data for all assessment indicators. The scores of the 12 experts and the corresponding cloud digital characteristics were added in Table 6, which improves the transparency of the expert evaluation process. The expert scores were determined based on the actual conditions of the case-study high-rise building and the field investigation results. Regarding the comparison with an existing method, we expanded the model comparison section. Specifically, we explained the correspondence between the indicators of the fire risk index method and the dimensions of Integrated Disaster Risk Theory. Disaster-inducing factors mainly correspond to the hazard dimension; passive and active fire-protection measures mainly correspond to vulnerability; and fire management and firefighting capability mainly correspond to emergency response and recovery capacity. We also normalized the FRI result to a 0–100 scale and compared it with the expectation value of the comprehensive cloud. The normalized FRI value was 71.58, while the expectation value obtained using the proposed method was 78.6933, with an absolute difference of 7.1133. Although the two methods differ in indicator structure, weighting method, and grade-determination procedure, they reached the same qualitative conclusion, indicating that the case-study building had a relatively low fire risk.

**Author action:** In the revised draft, we clarified the professional backgrounds and work experience of the 12 experts (lines 445-449), supplemented the original expert rating data for all evaluation indicators (Table 6), and demonstrated the differences between the two methods in the methodological comparison (lines 491-520).

**Reviewer#2, Concern # 4:** **There are many grammatical errors in English expressions and improper use of technical terms; incorrect typesetting of mathematical formulas, garbled characters; confused chart numbers, missing legends/units.**

**Author response:** We are grateful to the reviewer for pointing out these issues. We have checked and revised the English expressions, technical terms, mathematical formulas, figure and table numbering, legends, and units throughout the manuscript. In the revised manuscript, we improved the clarity and consistency of academic expressions, standardized the terminology related to high-rise building fire risk assessment, and corrected several inappropriate or inconsistent indicator names. We also checked the numbering and captions of figures and tables to ensure their consistency with the corresponding descriptions in the main text.

**Author action:** The manuscript was carefully proofread and revised for grammar, terminology, formula presentation, figure and table numbering, captions, and units. The indicator names were further checked against the revised indicator system, and the supporting information was reorganized to present the detailed calculation process more clearly.
