# Supplementary material for: Fire risk assessment for high-rise buildings based on Integrated Disaster Risk Theory
Source: PLoS One. 2026 Oct 5;21(10):e0358941. doi: 10.1371/journal.pone.0358941 (PMC13637918; doi:10.1371/journal.pone.0358941)
Supplement: Supplementary file 3 [file pone.0358941.s004.docx]

**Original Manuscript Number:** PONE-D-26-13643

**Original Article Title: “Fire risk assessment for high-rise buildings based on Integrated Disaster Risk Theory**”

Dear Editor,

We would like to express our sincere gratitude to you and the reviewers for the valuable time and constructive comments devoted to our manuscript (Manuscript Number: PONE-D-26-13643). The insightful suggestions have been instrumental in helping us significantly improve the quality of our work.

We have carefully considered all the comments and have revised the manuscript accordingly. Our point-by-point responses to each comment are detailed below.

We believe the manuscript is now strengthened and hope it meets the journal's standards for publication.

Thank you for your consideration.

Best regards,

Zonglai Liu et al.

**Reviewer#2, Concern # 1: The stability verification of the cloud similarity calculation is still incomplete. You now report that 4,000 cloud drops were generated and that the Grade II similarity (0.5898) is far higher than the adjacent grades, which is reassuring. Nevertheless, the forward cloud generator is a stochastic procedure, and a single run does not demonstrate reproducibility. Please repeat the similarity calculation a reasonable number of times (e.g., 10–30 independent runs) and report the mean and standard deviation of the similarity values for each grade, or at minimum show that the grade assignment is invariant across repeated runs. This can be added as a short paragraph in Section 3.2.3 or in the Supporting Information.**

**Author response:** Thank you for this valuable suggestion. We repeated the cloud similarity calculation independently 10 times while keeping the original assessment data, cloud parameters, and number of cloud drops (4,000 drops) unchanged. The mean values and standard deviations of the similarity values for each risk grade were calculated. The results show that the mean similarity values for Grades I–V were 0.0029, 0.5906, 0.0045, 0, and 0, respectively, with corresponding standard deviations of 0.0006, 0.0062, 0.0008, 0, and 0. The Grade II similarity remained consistently dominant in all repeated calculations, and the final risk grade assignment was unchanged as Grade II. These results demonstrate that the stochastic variation introduced by the forward cloud generator has a negligible influence on the final grade determination.

**Author action:** We have revised Section 3.2.3 of the main manuscript (Lines 475–478) and added the corresponding cloud similarity calculation results in the Supporting Information (Appendix Table A11).

**Reviewer#2, Concern # 2:** **The justification for the hyper-entropy setting He = 0.5 remains essentially an appeal to convention. Since He controls the "thickness" of the standard clouds and could in principle affect the similarity values near grade boundaries, I suggest adding a brief sensitivity analysis in which He is varied over a plausible range (e.g., 0.1–1.0) to show that the final grade determination is insensitive to this parameter. Given that your comprehensive cloud lies well inside the Grade II interval, I expect this analysis to be straightforward and to strengthen the robustness claim considerably.**

**Author response:** Thank you for this valuable suggestion. The hyper-entropy values of the standard clouds were varied from 0.1 to 1.0 with an interval of 0.1, while all other parameters, including the comprehensive cloud characteristics and cloud generation settings, remained unchanged. The results showed that although the similarity values varied slightly under different hyper-entropy settings, Grade II consistently exhibited the highest similarity among all risk grades. Specifically, the similarity values of Grade II ranged from 0.5818 to 0.5979, which remained substantially higher than those of the adjacent grades. Therefore, the final fire risk grade remained unchanged as Grade II throughout the sensitivity analysis. These results demonstrate that the proposed assessment model is robust against reasonable variations in the hyper-entropy parameter, and the selection of He=0.5 does not artificially determine the final assessment result.

**Author action:** We have revised Section 3.2.3 of the main manuscript (Line 478) and added the results of the hyper-entropy sensitivity analysis in the Supporting Information (Appendix Table A12).

**Reviewer#2, Concern # 3:** **Although the composition and experience of the 12 experts are now described, the scoring protocol itself is still insufficiently documented. Please clarify: (a) whether the experts scored independently or after group discussion; (b) whether a written scoring rubric or anchoring descriptions (e.g., what conditions correspond to a score of 60 versus 80 for a given indicator) was provided; and (c) some quantitative measure of inter-rater agreement, such as Kendall's W or the coefficient of variation per indicator. Table 6 shows non-trivial dispersion for several indicators (e.g., E3 ranges from 69 to 96), so a consistency statistic would help readers judge the reliability of the expert input.**

**Author response:** Thank you for this valuable comment. In this study, the 12 experts independently scored each indicator based on the actual conditions of the case building and their professional knowledge. Before scoring, the experts were provided with the definitions of all indicators and the corresponding risk-level intervals listed in Table 5. The independent scores were directly used as inputs for the cloud model to characterize the uncertainty and fuzziness of expert judgments. Regarding the inter-rater agreement analysis, we appreciate the reviewer’s suggestion. However, Kendall’s W or coefficient of variation was not adopted as a strict agreement measure in this study. The reason is that the expert panel consisted of specialists from different professional fields, and differences in their evaluations are expected because different experts may emphasize different aspects of high-rise building fire risk. These differences were considered meaningful information reflecting the diversity of expert knowledge rather than inconsistent judgments. The cloud model was specifically adopted to incorporate such randomness and fuzziness in expert evaluations.

**Author action:** We have revised Section 3.2 of the main manuscript (Lines 450-455).

**Reviewer#2, Concern # 4:** **The quantification basis for the newly added exposure indicators E4 (tangible assets) and E5 (intangible assets) needs elaboration. These are conceptually sound additions, but it is unclear what information the experts relied on when scoring them for a residential building of this type—was any estimate of property value, business interruption potential, or replacement cost made available to the panel? A sentence or two describing the evidence base for these two indicators would prevent the impression that they were scored purely impressionistically.**

**Author response:** Thank you for this valuable comment. For E4 (tangible assets), the experts considered the direct economic value associated with the building, including the estimated property value, construction investment cost, building scale, structural characteristics, and the potential direct losses of building components, equipment, facilities, and indoor property caused by fire. For E5 (intangible assets), considering that the case building is a residential high-rise building with commercial functions on the lower floors, the experts did not evaluate this indicator solely based on traditional business interruption losses. Instead, they considered the potential interruption of residential functions, impacts on ground-floor commercial services, social influence, recovery requirements, and possible changes in property value after similar fire events.

**Author action:** We have revised Section 2.1 of the main manuscript (Lines 205-207 , lines 209-211).

**Reviewer#2, Concern # 5:** **The comparison with the FRI method, while expanded, still overstates what the exercise demonstrates. The linear normalization of the FRI score (3.579 on a 1–5 scale mapped to 71.58) should be stated explicitly in the text, including the direction of the scale (i.e., confirmation that higher FRI values correspond to lower risk, so that the two scales are commensurable). Moreover, agreement between two expert-judgment-based methods applied by the same investigators to the same building constitutes consistency, not independent validation. I recommend softening the wording ("confirming the validity") in the Abstract and Conclusions to something like "supporting the plausibility/consistency of the proposed model," and acknowledging that both methods share partially overlapping subjective inputs.**

**Author response:** Thank you for this valuable comment. The linear normalization procedure of the FRI score was already described in the original manuscript (Lines 490–497), corresponding to Lines 547–549 in the revised manuscript. In the revised manuscript, we further clarified the meaning and direction of the FRI score, indicating that higher FRI scores correspond to better fire safety performance and lower fire risk levels. The FRI risk classification criteria were also added. In addition, the descriptions in the Abstract, Methods, and Model Comparison sections were revised to avoid overstating the validation capability of the proposed model. The comparison with the FRI method is now interpreted as supporting the plausibility and consistency of the proposed assessment framework.

**Author action:** We revised the relevant descriptions in the Abstract (Lines 28–29), Methods (Line 149-150), Model Comparison section (Lines 553–557), and Conclusions (Lines 577–578).

**Reviewer#2, Concern # 6: Section 3.3 remains rather generic. The value of an indicator-based model lies precisely in its diagnostic capability, yet the analysis discusses only dimension-level weights. Please link the discussion to the actual case results: for example, V7 (refuge floors and facilities, Ex = 67.5), V1 (proportion of elderly and children, Ex = 69.25), and E1 (population size, Ex = 69.83) received the lowest expert scores, which is notable given that more than half of the residents are reported to be elderly or under 14. Identifying these weak items and proposing correspondingly targeted measures would make the practical recommendations concrete rather than boilerplate. Related to this, please clarify the applicability of V7 to the case building: under Chinese code, refuge floors are mandated only for buildings exceeding 100 m, whereas the case building is 49.15 m; explain how experts were instructed to score an indicator that is not normatively required for the assessed object.**

**Author response:** Thank you for your valuable suggestion. In the revised manuscript, we have supplemented the indicator-level analysis based on the actual assessment results of the case-study building. Specifically, the indicators with relatively low expert evaluation values, including V7, V1, and E1, were identified and further discussed. The revised discussion explains the specific risk implications of these weak indicators and proposes targeted improvement measures. Regarding V7, we appreciate the reviewer’s comment on its applicability. The purpose of this indicator is not to evaluate only the mandatory installation of refuge floors, but to assess the overall refuge-related evacuation support capacity of high-rise buildings. For buildings below 100 m, such as the case-study building (49.15 m), experts were instructed to evaluate V7 mainly based on the availability, accessibility, and effectiveness of applicable refuge-related and auxiliary evacuation facilities. For buildings exceeding 100 m, both refuge floors and related refuge facilities should be considered. Therefore, the V7 indicator can be applied to different types of high-rise buildings by considering their specific structural characteristics and evacuation requirements. (the configuration and requirements of such escape apparatus are specified in GB 21976, Escape Apparatus for Building Fire).

**Author action:** We have revised Section 3.3 of the manuscript (lines 493–511).

**Reviewer#2, Concern # 7:** **There is an internal inconsistency in equation cross-referencing and a residual typesetting issue. In Section 2.2.3, the text states "Solving the linear system in Eq. (15)" when the linear system is actually Eq. (14). In addition, the specific game-theoretic combination coefficients (0.3571/0.6429 for the primary indices and 0.3122/0.6878 for the secondary indices) that you provided in the response letter should also appear in the manuscript or Supporting Information, since readers will not have access to the response document. Please also verify that all matrices in the SI are numbered consistently with the citations in the main text (Appendix Tables A1–A10 versus "Appendix Tables 1-5/1-10" as variously referenced).**

**Author response:** Thank you for this valuable comment. The incorrect cross-reference in Section 2.2.3 has been corrected, and the equation numbering has been carefully checked throughout the manuscript. Regarding the game-theoretic combination coefficients, the calculation procedure, relevant equations, and the input weight vectors obtained from FAHP and SEWM have already been provided in the manuscript and Supporting Information. Therefore, the combination coefficients can be directly derived according to the described method. In addition, the Supporting Information has been carefully checked, and the numbering of supplementary tables has been unified as Appendix Tables A1–A12 to ensure consistency with the citations in the main text.

**Author action:** We have corrected errors in Section 2.2.3 (line 352) and the Supporting Information.

**Reviewer#2, Concern # 8:** **The English has improved but still requires careful polishing. A few examples: "These dates point to the need..." (line 49) should read "These data point to..."; "Determination the standard cloud of assessment" should be "Determination of the standard cloud"; missing spaces after periods appear in several places (e.g., "related equipment. Meanwhile" around line 181); the descriptor "Fire risk is mediocre" in Table 5 is not idiomatic and should be "moderate"; and full-width punctuation and parentheses are mixed with half-width forms in Tables 1 and 2 and in several equations. The Conclusions also contain a sentence lacking terminal punctuation ("It reflects the specific characteristics of high-rise building fires"). A final round of professional language editing is recommended.**

**Author response:** Thank you for your valuable suggestion. We have carefully revised the manuscript and conducted a comprehensive language and formatting check. In addition, full-width and half-width punctuation marks have been standardized throughout the manuscript, and the overall English expression has been further polished.

**Author action:** The manuscript has been thoroughly proofread and revised accordingly.

**Reviewer#3, Concern # 1:** **Literature & innovation: Briefly compare your index framework with the commercial complex assessment paper in Discussion, highlight unique high-rise vertical evacuation & external rescue indicators to further distinguish incremental contributions; trim repetitive descriptions of disaster risk theory in Introduction and Section 2.1.**

**Author response:** Thank you for your valuable suggestion. In the revised manuscript, we further clarified the differences between the proposed index framework and previous fire risk assessment frameworks for large commercial buildings and shopping malls. Compared with previous studies, the proposed framework places greater emphasis on the specific evacuation and rescue characteristics of high-rise buildings, particularly vertical evacuation-related indicators and city-level external rescue capacity. These considerations better reflect the long vertical evacuation distances and greater dependence on professional rescue forces in high-rise building fires, thereby further distinguishing the incremental contribution of the proposed framework. In addition, we streamlined the descriptions of Integrated Disaster Risk Theory in the Introduction and Section 2.1 to reduce repetition.

**Author action:** We streamlined the description of Integrated Disaster Risk Theory in Section 2.1 (lines 158–163) and added a brief comparison with previous fire risk assessment frameworks for large commercial buildings and shopping malls in Section 3.3, “Analysis of the assessment results” (lines 512–517).

**Reviewer#3, Concern # 2:** **Calculation transparency: Add inter-rater consistency metric of 12 experts in SI; repeat cloud similarity calculation 1–2 times in appendix to prove stability; unify mathematical symbol format of FAHP compatibility index and game theory weight equations, fix garbled formulas.**

**Author response:** Thank you for your valuable suggestion. The 12 experts independently evaluated each indicator based on the actual conditions of the case building, the indicator definitions, and the corresponding risk-level intervals. Considering the different professional backgrounds of the experts, differences in their evaluations were regarded as meaningful information reflecting the diversity of expert knowledge rather than being treated solely as inconsistency. Therefore, Kendall’s W or the coefficient of variation was not adopted as a strict inter-rater agreement measure, while the randomness and fuzziness of the expert assessments were incorporated through the cloud model. In addition, the cloud similarity calculation was independently repeated 10 times under unchanged assessment data, cloud parameters, and number of cloud drops. The results showed that Grade II consistently had the highest similarity in all repeated calculations, with a mean similarity of 0.5906 and a standard deviation of 0.0062, confirming the stability of the final grade determination. The mathematical symbols and equation formats for the FAHP compatibility index and game-theoretic combination weighting were also carefully checked and standardized, and the garbled or inconsistent formulas were corrected.

**Author action:** We added the results of 10 independent repeated cloud similarity calculations in the Supporting Information (Appendix Table A11) and standardized the mathematical symbols and equation formats for the FAHP compatibility index and game-theoretic combination weighting.

**Reviewer#3, Concern # 3:** **Case & comparison: In model comparison section, add simple statistical analysis of score deviation between your cloud model and FRI method; briefly explain the limitation of single residential case and clearer multi-scenario future research outlook.**

**Author response:** Thank you for your valuable suggestion. In the Model Comparison section, we further quantified the deviation between the two assessment results. After normalization of the FRI score to a 0–100 scale, the FRI result was 71.58, compared with the comprehensive-cloud expectation value of 78.6933. The absolute deviation was 7.1133, corresponding to a relative deviation of approximately 9.04%. Although a numerical difference exists, both methods yielded the same qualitative conclusion of relatively low fire risk. In addition, we clarified the limitation associated with using a single high-rise residential building and further emphasized the need for validation using buildings with different functions, heights, regions, and management conditions in future research.

**Author action:** The comparison analysis has been supplemented with the statistical deviation analysis between the proposed cloud model and the FRI method. The limitations of the single case study and the future research prospects for multi-scenario applications have also been added to the revised manuscript.

**Reviewer#3, Concern # 4:** **Format & language: Full proofread English grammar and professional terms; revise inconsistent figure captions, table footnotes and missing unit labels; unify abbreviation definitions of all index dimensions at first occurrence.**

**Author response:** Thank you for your valuable suggestion. We have carefully proofread the revised manuscript to improve English grammar, academic expressions, and the consistency of professional terminology. In addition, we have checked and revised the figure captions, table descriptions, and unit labels throughout the manuscript where necessary.

**Author action:** The manuscript has been carefully proofread and revised for grammar, professional terminology, figure captions, table descriptions, and unit labels.

**Reviewer#3, Concern # 5:** **Supplementary material: Sort FAHP, entropy weight raw tables orderly in Supporting Information, add clear table titles for all appendix calculation data for easy review.**

**Author response:** Thank you for your valuable suggestion. The FAHP-related calculation tables (Appendix Tables A1–A5) and SEWM-related calculation tables (Appendix Tables A6–A10) have been arranged in a sequential order according to the indicator hierarchy. In addition, the titles of all appendix tables have been revised to improve clarity and allow readers to identify the corresponding calculation data more easily.

**Author action:** We reorganized the supplementary calculation tables and revised the titles of Appendix Tables A1–A12 in the Supporting Information. The FAHP, SEWM, cloud similarity calculation, and sensitivity analysis data are now presented in a clearer and more systematic order.
