# Supplementary material for: Fire risk assessment for high-rise buildings based on Integrated Disaster Risk Theory
Source: PLoS One. 2026 Oct 5;21(10):e0358941. doi: 10.1371/journal.pone.0358941 (PMC13637918; doi:10.1371/journal.pone.0358941)
Supplement: Peer review history

# Peer review: Decision Letter 0

**DOI:** 10.1371/journal.pone.0358941.r001

**Authors:** Yang (Jack) Lu

29 Apr 2026

Dear Dr. liu,

Thank you for submitting your manuscript to PLOS ONE. After careful consideration, we feel that it has merit but does not fully meet PLOS ONE’s publication criteria as it currently stands. Therefore, we invite you to submit a revised version of the manuscript that addresses the points raised during the review process.

Please submit your revised manuscript by Jun 13 2026 11:59PM. If you will need more time than this to complete your revisions, please reply to this message or contact the journal office at plosone@plos.org. When you're ready to submit your revision, log on to https://www.editorialmanager.com/pone/ and select the 'Submissions Needing Revision' folder to locate your manuscript file.

- A letter that responds to each point raised by the academic editor and reviewer(s). You should upload this letter as a separate file labeled 'Response to Reviewers'.
- A marked-up copy of your manuscript that highlights changes made to the original version. You should upload this as a separate file labeled 'Revised Manuscript with Track Changes'.
- An unmarked version of your revised paper without tracked changes. You should upload this as a separate file labeled 'Manuscript'.

If you would like to make changes to your financial disclosure, please include your updated statement in your cover letter. Guidelines for resubmitting your figure files are available below the reviewer comments at the end of this letter.

If applicable, we recommend that you deposit your laboratory protocols in protocols.io to enhance the reproducibility of your results. Protocols.io assigns your protocol its own identifier (DOI) so that it can be cited independently in the future. For instructions see: https://journals.plos.org/plosone/s/submission-guidelines#loc-laboratory-protocols. Additionally, PLOS ONE offers an option for publishing peer-reviewed Lab Protocol articles, which describe protocols hosted on protocols.io. Read more information on sharing protocols at https://plos.org/protocols?utm_medium=editorial-email&utm_source=authorletters&utm_campaign=protocols.

As the corresponding author, your ORCID iD is verified in the submission system and will appear in the published article. PLOS supports the use of ORCID, and we encourage all coauthors to register for an ORCID iD and use it as well. Please encourage your coauthors to verify their ORCID iD within the submission system before final acceptance, as unverified ORCID iDs will not appear in the published article. *Only*  the individual author can complete the verification step; PLOS staff *cannot*  verify ORCID iDs on behalf of authors.

We look forward to receiving your revised manuscript.

Kind regards,

Yang (Jack) Lu, PhD

Journal Requirements:

When submitting your revision, we need you to address these additional requirements.

1. Please ensure that your manuscript meets PLOS ONE's style requirements, including those for file naming. The PLOS ONE style templates can be found at

https://journals.plos.org/plosone/s/file?id=wjVg/PLOSOne_formatting_sample_main_body.pdf and https://journals.plos.org/plosone/s/file?id=ba62/PLOSOne_formatting_sample_title_authors_affiliations.pdf

2. Please note that PLOS One has specific guidelines on code sharing for submissions in which author-generated code underpins the findings in the manuscript. In these cases, we expect all author-generated code to be made available without restrictions upon publication of the work. Please review our guidelines at https://journals.plos.org/plosone/s/materials-and-software-sharing#loc-sharing-code and ensure that your code is shared in a way that follows best practice and facilitates reproducibility and reuse.

3. Thank you for stating the following in your Competing Interests section:

[NO authors have competing interests].

Please complete your Competing Interests on the online submission form to state any Competing Interests. If you have no competing interests, please state "The authors have declared that no competing interests exist.", as detailed online in our guide for authors at http://journals.plos.org/plosone/s/submit-now

This information should be included in your cover letter; we will change the online submission form on your behalf.

4. We note that your Data Availability Statement is currently as follows: [All relevant data are within the manuscript and its Supporting Information files]

Please confirm at this time whether or not your submission contains all raw data required to replicate the results of your study. Authors must share the “minimal data set” for their submission. PLOS defines the minimal data set to consist of the data required to replicate all study findings reported in the article, as well as related metadata and methods (https://journals.plos.org/plosone/s/data-availability#loc-minimal-data-set-definition).

For example, authors should submit the following data:

- The values behind the means, standard deviations and other measures reported;

- The values used to build graphs;

- The points extracted from images for analysis.

Authors do not need to submit their entire data set if only a portion of the data was used in the reported study.

If your submission does not contain these data, please either upload them as Supporting Information files or deposit them to a stable, public repository and provide us with the relevant URLs, DOIs, or accession numbers. For a list of recommended repositories, please see https://journals.plos.org/plosone/s/recommended-repositories.

If there are ethical or legal restrictions on sharing a de-identified data set, please explain them in detail (e.g., data contain potentially sensitive information, data are owned by a third-party organization, etc.) and who has imposed them (e.g., an ethics committee). Please also provide contact information for a data access committee, ethics committee, or other institutional body to which data requests may be sent. If data are owned by a third party, please indicate how others may request data access.

5. PLOS requires an ORCID iD for the corresponding author in Editorial Manager on papers submitted after December 6th, 2016. Please ensure that you have an ORCID iD and that it is validated in Editorial Manager. To do this, go to ‘Update my Information’ (in the upper left-hand corner of the main menu), and click on the Fetch/Validate link next to the ORCID field. This will take you to the ORCID site and allow you to create a new iD or authenticate a pre-existing iD in Editorial Manager.

6. Thank you for stating the following in the Competing Interests section:

[NO authors have competing interests].

We note that one or more of the authors are employed by a commercial company: Jilin Heyuan Fire Engineering Co., Ltd.

1. Please provide an amended Funding Statement declaring this commercial affiliation, as well as a statement regarding the Role of Funders in your study. If the funding organization did not play a role in the study design, data collection and analysis, decision to publish, or preparation of the manuscript and only provided financial support in the form of authors' salaries and/or research materials, please review your statements relating to the author contributions, and ensure you have specifically and accurately indicated the role(s) that these authors had in your study. You can update author roles in the Author Contributions section of the online submission form.

Please also include the following statement within your amended Funding Statement.

“The funder provided support in the form of salaries for authors [insert relevant initials], but did not have any additional role in the study design, data collection and analysis, decision to publish, or preparation of the manuscript. The specific roles of these authors are articulated in the ‘author contributions’ section.”

If your commercial affiliation did play a role in your study, please state and explain this role within your updated Funding Statement.

2. Please also provide an updated Competing Interests Statement declaring this commercial affiliation along with any other relevant declarations relating to employment, consultancy, patents, products in development, or marketed products, etc.

Within your Competing Interests Statement, please confirm that this commercial affiliation does not alter your adherence to all PLOS ONE policies on sharing data and materials by including the following statement: "This does not alter our adherence to PLOS ONE policies on sharing data and materials.” (as detailed online in our guide for authors http://journals.plos.org/plosone/s/competing-interests) . If this adherence statement is not accurate and there are restrictions on sharing of data and/or materials, please state these. Please note that we cannot proceed with consideration of your article until this information has been declared.

Please include both an updated Funding Statement and Competing Interests Statement in your cover letter. We will change the online submission form on your behalf.

7. We note that Figure 1 in your submission contains images which may be copyrighted. All PLOS content is published under the Creative Commons Attribution License (CC BY 4.0), which means that the manuscript, images, and Supporting Information files will be freely available online, and any third party is permitted to access, download, copy, distribute, and use these materials in any way, even commercially, with proper attribution. For more information, see our copyright guidelines: http://journals.plos.org/plosone/s/licenses-and-copyright.

We require you to either (1) present written permission from the copyright holder to publish these figures specifically under the CC BY 4.0 license, or (2) remove the figures from your submission:

1. You may seek permission from the original copyright holder of Figure 1 to publish the content specifically under the CC BY 4.0 license.

We recommend that you contact the original copyright holder with the Content Permission Form (http://journals.plos.org/plosone/s/file?id=7c09/content-permission-form.pdf) and the following text:

“I request permission for the open-access journal PLOS ONE to publish XXX under the Creative Commons Attribution License (CCAL) CC BY 4.0 (http://creativecommons.org/licenses/by/4.0/). Please be aware that this license allows unrestricted use and distribution, even commercially, by third parties. Please reply and provide explicit written permission to publish XXX under a CC BY license and complete the attached form.”

Please upload the completed Content Permission Form or other proof of granted permissions as an "Other" file with your submission.

In the figure caption of the copyrighted figure, please include the following text: “Reprinted from [ref] under a CC BY license, with permission from [name of publisher], original copyright [original copyright year].”

2. If you are unable to obtain permission from the original copyright holder to publish these figures under the CC BY 4.0 license or if the copyright holder’s requirements are incompatible with the CC BY 4.0 license, please either i) remove the figure or ii) supply a replacement figure that complies with the CC BY 4.0 license. Please check copyright information on all replacement figures and update the figure caption with source information. If applicable, please specify in the figure caption text when a figure is similar but not identical to the original image and is therefore for illustrative purposes only.

If the reviewer comments include a recommendation to cite specific previously published works, please review and evaluate these publications to determine whether they are relevant and should be cited. There is no requirement to cite these works unless the editor has indicated otherwise.

[Note: HTML markup is below. Please do not edit.]

Reviewers' comments:

Reviewer's Responses to Questions

**Comments to the Author**

1. Is the manuscript technically sound, and do the data support the conclusions?

Reviewer #1: Partly

Reviewer #2: Partly

**********

2. Has the statistical analysis been performed appropriately and rigorously?

Reviewer #1: Yes

Reviewer #2: No

**********

3. Have the authors made all data underlying the findings in their manuscript fully available?

The PLOS Data policy

Reviewer #1: Yes

Reviewer #2: Yes

**********

4. Is the manuscript presented in an intelligible fashion and written in standard English?

Reviewer #1: Yes

Reviewer #2: No

**********

Reviewer #1: The manuscript introduces comprehensive disaster risk theory into high-rise building fire risk assessment, combines game theory combination empowerment and cloud model to build an assessment model, and uses a high-rise building in Changchun as a case for verification. The research topic is in line with the actual needs of urban public safety. However, there are still some areas for improvement in the manuscript.

1. The innovation points are vaguely stated, and the incremental contribution compared to existing methods is not clearly explained.

2. Related research should be more comprehensive, such as Potential of large language models in blockchain-based supply chain finance[J]. Enterprise Information Systems, 2025: 2541199. and FIRE RISK ASSESSMENT FOR LARGE COMMERCI`AL COMPLEXES BASED ON INTEGRATED DISASTER RISK THEORY USING GAME-THEORETIC COMBINATION WEIGHTING AND THE CLOUD MODEL. International Journal of Infrastructure Research and Management, 2024, 12(1), 44-71.

3. The exposure dimension only has 3 indicators, which lack sufficient coverage and lacks key items such as economic losses and evacuation channels.

4. The FAHP consistency test results are not given, and the validity of the expert judgment matrix cannot be judged.

5. The cloud similarity calculation does not specify the number of repetitions and stability verification, and the results are not credible enough.

Reviewer #2: 1. The comprehensive disaster risk theory is directly copied to the fire of high-rise buildings. The adaptability of the theory and the formation mechanism of the fire has not been proved, and the dimensional division is mechanical and untargeted. It is recommended to supplement a paragraph to argue how the four dimensions of the comprehensive disaster risk theory can be adapted to man-made fires in high-rise buildings, and clarify the corresponding logic of "danger - fire triggers, exposure - personnel and property, vulnerability - building fire resistance, emergency response capacity - internal and external rescue".

2. FAHP does not clearly show the basis for the calculation results of the compatibility index and the consistency test threshold α; the structural entropy weighting method does not disclose the expert grouping method and the original scoring data; the solution process of the linear equation system of the combination weight of the game theory and the coefficient normalisation steps are missing; the cloud model superentropy He fixed takes 0.5 Without any basis, the stability of cloud similarity calculation has not been verified. It is recommended to supplement the specific value of the compatibility index I (A,W*) of FAHP to explain the reason for the selection of α=0.1; clarify the setting basis of He=0.5 in the cloud model, and add 1-2 cloud similarity repeat tests to verify the stability; and complete the linear square of the weight of game theory. The result of solving the matrix and coefficient of the program group.

3. Only one residential high-rise building in Changchun is used as a case, and there is no multi-building type and multi-regional verification; 12 experts did not disclose the professional background, scoring standards and scoring consistency; the comparison with the existing method is only at the result level, and there is no process alignment and statistical inspection. It is recommended to clarify the field (fire engineering / construction safety / emergency management) and years of practice of the 12 experts; supplement the details of the field survey of fire hazards of case construction, improve the original data table of expert scoring; add indicator alignment instructions and statistical analysis of result differences when comparing and verification.

4. There are many grammatical errors in English expressions and improper use of technical terms; incorrect typesetting of mathematical formulas, garbled characters; confused chart numbers, missing legends/units.

**********

what does this mean?). If published, this will include your full peer review and any attached files.

If you choose “no”, your identity will remain anonymous but your review may still be made public.

**Do you want your identity to be public for this peer review?**  For information about this choice, including consent withdrawal, please see our Privacy Policy

Reviewer #1: No

Reviewer #2: No

**********

To ensure your figures meet our technical requirements, please review our figure guidelines: https://journals.plos.org/plosone/s/figures

You may also use PLOS’s free figure tool, NAAS, to help you prepare publication quality figures: https://journals.plos.org/plosone/s/figures#loc-tools-for-figure-preparation.

NAAS will assess whether your figures meet our technical requirements by comparing each figure against our figure specifications.

# Author comment: Author response to Decision Letter 1

**DOI:** 10.1371/journal.pone.0358941.r002

1 Jul 2026

Original Manuscript Number: PONE-D-26-13643

Original Article Title: “Fire risk assessment for high-rise buildings based on Integrated Disaster Risk Theory”

Dear Editor,

We would like to express our sincere gratitude to you and the reviewers for the valuable time and constructive comments devoted to our manuscript (Manuscript Number: PONE-D-26-13643). The insightful suggestions have been instrumental in helping us significantly improve the quality of our work.

We have carefully considered all the comments and have revised the manuscript accordingly. Our point-by-point responses to each comment are detailed below.

We believe the manuscript is now strengthened and hope it meets the journal's standards for publication.

Thank you for your consideration.

Best regards,

Zonglai Liu et al.

Reviewer#1, Concern # 1: The innovation points are vaguely stated, and the incremental contribution compared to existing methods is not clearly explained.

Author response: Thank you for your constructive feedback. The main objective of this study is to construct a fire risk assessment indicator system specifically applicable to high-rise buildings. Compared with ordinary buildings, high-rise buildings face greater challenges in evacuation, fire suppression, and rescue operations after a fire occurs. Therefore, the emergency response and recovery capacity of high-rise buildings requires more comprehensive consideration. In the revised manuscript, this dimension is strengthened by incorporating both building-level internal fire safety capacity and city-level external fire and rescue capacity. Moreover, the compatibility between firefighting equipment and building characteristics is included to reflect the adaptability of professional rescue resources to high-rise building fire scenarios.

Author action: We have revised the abstract (lines 21-23, 29-31), introduction (lines 87-116), and conclusion (lines 525-547) in the main text.________________________________________

Reviewer#1, Concern # 2: Related research should be more comprehensive, such as Potential of large language models in blockchain-based supply chain finance[J]. Enterprise Information Systems, 2025: 2541199. and FIRE RISK ASSESSMENT FOR LARGE COMMERCI`AL COMPLEXES BASED ON INTEGRATED DISASTER RISK THEORY USING GAME-THEORETIC COMBINATION WEIGHTING AND THE CLOUD MODEL. International Journal of Infrastructure Research and Management, 2024, 12(1), 44-71.

Author response: Thank you for your valuable suggestion. We have carefully reviewed and fully considered the relevant studies suggested by the reviewer. Among them, the study on fire risk assessment for large commercial complexes is highly relevant to the present work. However, the research object and the logic of indicator construction in our study are clearly different from those of that study. Previous research mainly focused on large commercial complexes, such as shopping malls and mixed-use commercial buildings, whose major fire risk characteristics include high occupant density, complex commercial functions, high fire load, and long internal travel distances. In contrast, the present study focuses specifically on fire risk assessment for high-rise buildings. High-rise buildings face more prominent challenges in vertical evacuation, firefighting at height, smoke spread through vertical shafts, external rescue accessibility, and dependence on professional fire and rescue forces. Therefore, the main contribution of this study is not the simple reuse of existing assessment methods, but the reconstruction of a more targeted fire risk assessment index system according to the specific fire risk characteristics of high-rise buildings. In the revised manuscript, we further adjusted and improved the indicator system so that it can better reflect the risk characteristics of high-rise building fire scenarios. These revisions help distinguish the present study from previous commercial-complex-oriented assessment frameworks and improve the applicability of the indicator system to high-rise building fire risk assessment.

Author action: We revised the index section of the paper to reflect the differences from previous studies (lines 154-252). The revised index are shown in Table 1:

Table 1. High-rise Building Fire Risk Assessment Index System.

HRFRI Factor Index

High-rise Building Fire Risk Assessment Index System Hazard （H） Occupant activity intensity （H1）

Electrical facilities（H2）

Gas facilities（H3）

External environment（H4）

Indoor fire load(H5)

Building thermal insulation and exterior wall decoration（H6）

Exposure（E） Population size（E1）

Building functional complexity（E2）

Evacuation facility capacity（E3）

Tangible assets of the building(E4)

Intangible assets of the building (E5)

Vulnerability（V） Proportion of elderly and children（V1）

Building height（V2）

Building service life（V3）

Fire compartment（V4）

Fire-resistance rating（V5）

Evacuation routes and evacuation guidance facilities（V6）

Refuge floors and refuge facilities（V7）

Water-supply facilities（V8）

Automatic fire alarm system（V9）

Automatic sprinkler system（V10）

Ventilation and smoke exhaust system（V11）

Emergency response and recovery capacity（C） Fire management system（C1）

Fire-safety inspection（C2）

Fire-facility maintenance（C3）

Construction status of micro fire stations（C4）

Comprehensive rescue capacity of the local fire station（C5）

Firefighting response time（C6）

Compatibility between firefighting equipment and building characteristics （C7）

________________________________________

Reviewer#1, Concern # 3: The exposure dimension only has 3 indicators, which lack sufficient coverage and lacks key items such as economic losses and evacuation channels.

Author response: Thank you for your constructive comment. We agree that the exposure dimension in the original manuscript was not sufficiently comprehensive and did not fully cover the disaster-bearing bodies that may be directly affected after a high-rise building fire occurs. In disaster risk research, exposure mainly refers to the people and property at risk that are located within the potential impact range of a hazard. Therefore, in the revised manuscript, we expanded and refined the exposure dimension according to the characteristics of high-rise building fire scenarios. The revised exposure dimension includes five indicators from four aspects: human exposure, functional exposure, evacuation exposure, and property exposure. Population size within the building (E1) is used to reflect the number of occupants who may be threatened in the event of a fire. Building functional complexity (E2) is used to reflect the coupling of fire risk among different functional spaces. The newly added evacuation facility capacity (E3) is used to reflect the ability of stairwells, evacuation corridors, safety exits, and other evacuation facilities to meet occupant evacuation demand. In addition, tangible assets of the building (E4) and intangible assets of the building (E5) were added to characterize potential direct and indirect economic losses, respectively.

Author action: We have revised the exposure-related assessment index and provided an explanation of them (lines 191-211).

________________________________________

Reviewer#1, Concern # 4: The FAHP consistency test results are not given, and the validity of the expert judgment matrix cannot be judged.

Author response: Thank you for your constructive comment. In the revised manuscript, we have supplemented the consistency test results of the FAHP judgment matrices.

Author action: We have added all the results obtained using FAHP to Appendix Tables 1-5 of the supporting information.

________________________________________

Reviewer#1, Concern # 5: The cloud similarity calculation does not specify the number of repetitions and stability verification, and the results are not credible enough.

Author response: Thank you for your valuable suggestion. We agree that the original manuscript did not provide sufficient details on the cloud similarity calculation process and its results, which may have affected the reliability of the assessment results. Therefore, in the revised manuscript, we supplemented the number of cloud drops used in the cloud similarity calculation. Specifically, 4,000 cloud drops were generated for each similarity calculation. In addition, we clearly reported the similarity results between the comprehensive cloud and the five standard-grade clouds, namely Grade V: 0, Grade IV: 0, Grade III: 0.0046, Grade II: 0.5898, and Grade I: 0.0039. The results show that the comprehensive cloud had the highest similarity with the Grade II standard cloud; therefore, the fire risk grade of the project was determined as Grade II.

Author action: In section 3.2.3 of the main text, we supplemented the calculated number of cloud droplets (4000) and the calculation results of cloud similarity (V:0, IV:0, III:0.0046, II:0.5898, I:0.0039) to enhance the reliability of the results.

________________________________________

Reviewer#2, Concern # 1: The comprehensive disaster risk theory is directly copied to the fire of high-rise buildings. The adaptability of the theory and the formation mechanism of the fire has not been proved, and the dimensional division is mechanical and untargeted. It is recommended to supplement a paragraph to argue how the four dimensions of the comprehensive disaster risk theory can be adapted to man-made fires in high-rise buildings, and clarify the corresponding logic of "danger - fire triggers, exposure - personnel and property, vulnerability - building fire resistance, emergency response capacity - internal and external rescue".

Author response: Thank you for your constructive comment. We agree that the original manuscript did not sufficiently explain the applicability of Integrated Disaster Risk Theory to high-rise building fires, which may have made the dimensional division appear somewhat mechanical. In the revised manuscript, we supplemented the relevant explanation to further clarify why high-rise building fires can be incorporated into the framework of Integrated Disaster Risk Theory and how the four dimensions correspond to the fire risk formation mechanism. We consider building fires to be both urban safety accidents and emergency events. From the perspective of disaster science, they can be regarded as a type of human-induced disaster. Therefore, this study understands high-rise building fire risk as the result of the combined effects of fire-inducing factors, disaster-bearing bodies, the vulnerability of disaster-bearing bodies, and emergency response and recovery capacity. Within this framework, hazard mainly corresponds to the inducing conditions for fire occurrence and development; exposure mainly corresponds to the people and property that may be affected by a fire; vulnerability mainly corresponds to the susceptibility of occupants and buildings to damage under fire conditions; and emergency response and recovery capacity corresponds to the internal and external capacity for disaster prevention, resistance, emergency response, and recovery.

Author action: We have provided a detailed description of the application of the theory in Section 2.1 of the revised manuscript (lines 154-252).

________________________________________

Reviewer#2, Concern # 2: FAHP does not clearly show the basis for the calculation results of the compatibility index and the consistency test threshold α; the structural entropy weighting method does not disclose the expert grouping method and the original scoring data; the solution process of the linear equation system of the combination weight of the game theory and the coefficient normalisation steps are missing; the cloud model superentropy He fixed takes 0.5 Without any basis, the stability of cloud similarity calculation has not been verified. It is recommended to supplement the specific value of the compatibility index I (A,W*) of FAHP to explain the reason for the selection of α=0.1; clarify the setting basis of He=0.5 in the cloud model, and add 1-2 cloud similarity repeat tests to verify the stability; and complete the linear square of the weight of game theory. The result of solving the matrix and coefficient of the program group.

Author response: Thank you for the reviewer’s careful reading and constructive comments. In response to these concerns, we have made corresponding supplements in the revised manuscript and the Supporting Information. First, for the FAHP method, we supplemented the fuzzy complementary judgment matrices and the specific values of the compatibility index I(A,W*) in the Supporting Information. The consistency-test threshold was set as α = 0.1 because this value is commonly adopted in the consistency test of fuzzy complementary judgment matrices, and a smaller α indicates a stricter consistency requirement. Second, for the structural entropy weight method, the 12 experts were divided into four groups according to their professional backgrounds, and the original ranking matrices were added to the Supporting Information. Third, for the cloud model, we specified the number of cloud drops used in the cloud similarity calculation and provided the similarity values between the comprehensive cloud and the five standard clouds. The hyper-entropy parameter He was set to 0.5 following the commonly used setting in cloud-model-based risk assessment studies. This parameter is used to characterize the uncertainty of entropy in the standard evaluation clouds. In addition, the similarity corresponding to Grade II was substantially higher than those of the other grades, indicating that the assessment result was not located near the boundary between adjacent risk grades and that the final grade determination was relatively stable under the adopted calculation setting. Finally, regarding the game-theoretic combination weighting method, we agree that the solution process of the linear equation system and the coefficient normalization should be clarified. To maintain the readability and conciseness of the manuscript, the detailed numerical solution process was not expanded in the main text. The linear equation system of the primary index: , the normalized coefficients are 0.3571 and 0.6429, respectively, and the second-order index linear equation system ,the normalized coefficients are 0.3122 and 0.6878, respectively.

Author action: We have added specific calculations for FAHP and SEWM in the revised draft (Appendix Table 1-10 for Supporting information). In section 3.2.3 of the main text, we supplemented the calculated number of cloud droplets (4000) and the calculation results of cloud similarity (V:0, IV:0, III:0.0046, II:0.5898, I:0.0039) to enhance the reliability of the results.________________________________________

Reviewer#2, Concern # 3: Only one residential high-rise building in Changchun is used as a case, and there is no multi-building type and multi-regional verification; 12 experts did not disclose the professional background, scoring standards and scoring consistency; the comparison with the existing method is only at the result level, and there is no process alignment and statistical inspection. It is recommended to clarify the field (fire engineering / construction safety / emergency management) and years of practice of the 12 experts; supplement the details of the field survey of fire hazards of case construction, improve the original data table of expert scoring; add indicator alignment instructions and statistical analysis of result differences when comparing and verification.

Author response: Thank you for this valuable comment. In the revised manuscript, we clarified the professional background and working experience of the 12 experts. All invited experts had more than three years of professional experience in fire safety, emergency management, or building fire-protection operations. The expert panel consisted of three university researchers specializing in safety-related fields, three frontline firefighters from fire and rescue departments, three professionals from third-party fire safety institutions, and three fire-facility operators from property management companies. In addition, we supplemented the original expert scoring data for all assessment indicators. The scores of the 12 experts and the corresponding cloud digital characteristics were added in Table 6, which improves the transparency of the expert evaluation process. The expert scores were determined based on the actual conditions of the case-study high-rise building and the field investigation results. Regarding the comparison with an existing method, we expanded the model comparison section. Specifically, we explained the correspondence between the indicators of the fire risk index method and the dimensions of Integrated Disaster Risk Theory. Disaster-inducing factors mainly correspond to the hazard dimension; passive and active fire-protection measures mainly correspond to vulnerability; and fire management and firefighting capability mainly correspond to emergency response and recovery capacity. We also normalized the FRI result to a 0–100 scale and compared it with the expectation value of the comprehensive cloud. The normalized FRI value was 71.58, while the expectation value obtained using the proposed method was 78.6933, with an absolute difference of 7.1133. Although the two methods differ in indicator structure, weighting method, and grade-determination procedure, they reached the same qualitative conclusion, indicating that the case-study building had a relatively low fire risk.

Author action: In the revised draft, we clarified the professional backgrounds and work experience of the 12 experts (lines 445-449), supplemented the original expert rating data for all evaluation indicators (Table 6), and demonstrated the differences between the two methods in the methodological comparison (lines 491-520).

________________________________________

Reviewer#2, Concern # 4: There are many grammatical errors in English expressions and improper use of technical terms; incorrect typesetting of mathematical formulas, garbled characters; confused chart numbers, missing legends/units.

Author response: We are grateful to the reviewer for pointing out these issues. We have checked and revised the English expressions, technical terms, mathematical formulas, figure and table numbering, legends, and units throughout the manuscript. In the revised manuscript, we improved the clarity and consistency of academic expressions, standardized the terminology related to high-rise building fire risk assessment, and corrected several inappropriate or inconsistent indicator names. We also checked the numbering and captions of figures and tables to ensure their consistency with the corresponding descriptions in the main text.

Author action: The manuscript was carefully proofread and revised for grammar, terminology, formula presentation, figure and table numbering, captions, and units. The indicator names were further checked against the revised indicator system, and the supporting information was reorganized to present the detailed calculation process more clearly.________________________________________

Attachment Submitted filename: Response to Reviewers.docx

# Peer review: Decision Letter 1

**DOI:** 10.1371/journal.pone.0358941.r003

**Authors:** Yang (Jack) Lu

16 Jul 2026

Dear Dr. liu,

Thank you for submitting your manuscript to PLOS ONE. After careful consideration, we feel that it has merit but does not fully meet PLOS ONE’s publication criteria as it currently stands. Therefore, we invite you to submit a revised version of the manuscript that addresses the points raised during the review process.

Please submit your revised manuscript by Aug 30 2026 11:59PM. If you will need more time than this to complete your revisions, please reply to this message or contact the journal office at plosone@plos.org. When you're ready to submit your revision, log on to https://www.editorialmanager.com/pone/ and select the 'Submissions Needing Revision' folder to locate your manuscript file.

Please include the following items when submitting your revised manuscript:

- A letter that responds to each point raised by the academic editor and reviewer(s). You should upload this letter as a separate file labeled 'Response to Reviewers'.
- A marked-up copy of your manuscript that highlights changes made to the original version. You should upload this as a separate file labeled 'Revised Manuscript with Track Changes'.
- An unmarked version of your revised paper without tracked changes. You should upload this as a separate file labeled 'Manuscript'.

If you would like to make changes to your financial disclosure, please include your updated statement in your cover letter. Guidelines for resubmitting your figure files are available below the reviewer comments at the end of this letter.

If applicable, we recommend that you deposit your laboratory protocols in protocols.io to enhance the reproducibility of your results. Protocols.io assigns your protocol its own identifier (DOI) so that it can be cited independently in the future. For instructions see: https://journals.plos.org/plosone/s/submission-guidelines#loc-laboratory-protocols. Additionally, PLOS ONE offers an option for publishing peer-reviewed Lab Protocol articles, which describe protocols hosted on protocols.io. Read more information on sharing protocols at https://plos.org/protocols?utm_medium=editorial-email&utm_source=authorletters&utm_campaign=protocols.

As the corresponding author, your ORCID iD is verified in the submission system and will appear in the published article. PLOS supports the use of ORCID, and we encourage all coauthors to register for an ORCID iD and use it as well. Please encourage your coauthors to verify their ORCID iD within the submission system before final acceptance, as unverified ORCID iDs will not appear in the published article. Only the individual author can complete the verification step; PLOS staff cannot verify ORCID iDs on behalf of authors.

We look forward to receiving your revised manuscript.

Kind regards,

Yang (Jack) Lu, PhD

Academic Editor

PLOS One

Journal Requirements:

If the reviewer comments include a recommendation to cite specific previously published works, please review and evaluate these publications to determine whether they are relevant and should be cited. There is no requirement to cite these works unless the editor has indicated otherwise.

Please review your reference list to ensure that it is complete and correct. If you have cited papers that have been retracted, please include the rationale for doing so in the manuscript text, or remove these references and replace them with relevant current references. Any changes to the reference list should be mentioned in the rebuttal letter that accompanies your revised manuscript. If you need to cite a retracted article, indicate the article’s retracted status in the References list and also include a citation and full reference for the retraction notice.

[Note: HTML markup is below. Please do not edit.]

Reviewers' comments:

Reviewer's Responses to Questions

**Comments to the Author**

Reviewer #2: (No Response)

Reviewer #3: (No Response)

**********

2. Is the manuscript technically sound, and do the data support the conclusions?

Reviewer #2: Partly

Reviewer #3: Yes

**********

3. Has the statistical analysis been performed appropriately and rigorously?

Reviewer #2: No

Reviewer #3: Yes

**********

4. Have the authors made all data underlying the findings in their manuscript fully available?

The PLOS Data policy

Reviewer #2: Yes

Reviewer #3: No

**********

5. Is the manuscript presented in an intelligible fashion and written in standard English?

Reviewer #2: No

Reviewer #3: Yes

**********

Reviewer #2: 1. The stability verification of the cloud similarity calculation is still incomplete. You now report that 4,000 cloud drops were generated and that the Grade II similarity (0.5898) is far higher than the adjacent grades, which is reassuring. Nevertheless, the forward cloud generator is a stochastic procedure, and a single run does not demonstrate reproducibility. Please repeat the similarity calculation a reasonable number of times (e.g., 10–30 independent runs) and report the mean and standard deviation of the similarity values for each grade, or at minimum show that the grade assignment is invariant across repeated runs. This can be added as a short paragraph in Section 3.2.3 or in the Supporting Information.

2. The justification for the hyper-entropy setting He = 0.5 remains essentially an appeal to convention. Since He controls the "thickness" of the standard clouds and could in principle affect the similarity values near grade boundaries, I suggest adding a brief sensitivity analysis in which He is varied over a plausible range (e.g., 0.1–1.0) to show that the final grade determination is insensitive to this parameter. Given that your comprehensive cloud lies well inside the Grade II interval, I expect this analysis to be straightforward and to strengthen the robustness claim considerably.

3. Although the composition and experience of the 12 experts are now described, the scoring protocol itself is still insufficiently documented. Please clarify: (a) whether the experts scored independently or after group discussion; (b) whether a written scoring rubric or anchoring descriptions (e.g., what conditions correspond to a score of 60 versus 80 for a given indicator) was provided; and (c) some quantitative measure of inter-rater agreement, such as Kendall's W or the coefficient of variation per indicator. Table 6 shows non-trivial dispersion for several indicators (e.g., E3 ranges from 69 to 96), so a consistency statistic would help readers judge the reliability of the expert input.

4. The quantification basis for the newly added exposure indicators E4 (tangible assets) and E5 (intangible assets) needs elaboration. These are conceptually sound additions, but it is unclear what information the experts relied on when scoring them for a residential building of this type—was any estimate of property value, business interruption potential, or replacement cost made available to the panel? A sentence or two describing the evidence base for these two indicators would prevent the impression that they were scored purely impressionistically.

5. The comparison with the FRI method, while expanded, still overstates what the exercise demonstrates. The linear normalization of the FRI score (3.579 on a 1–5 scale mapped to 71.58) should be stated explicitly in the text, including the direction of the scale (i.e., confirmation that higher FRI values correspond to lower risk, so that the two scales are commensurable). Moreover, agreement between two expert-judgment-based methods applied by the same investigators to the same building constitutes consistency, not independent validation. I recommend softening the wording ("confirming the validity") in the Abstract and Conclusions to something like "supporting the plausibility/consistency of the proposed model," and acknowledging that both methods share partially overlapping subjective inputs.

6. Section 3.3 remains rather generic. The value of an indicator-based model lies precisely in its diagnostic capability, yet the analysis discusses only dimension-level weights. Please link the discussion to the actual case results: for example, V7 (refuge floors and facilities, Ex = 67.5), V1 (proportion of elderly and children, Ex = 69.25), and E1 (population size, Ex = 69.83) received the lowest expert scores, which is notable given that more than half of the residents are reported to be elderly or under 14. Identifying these weak items and proposing correspondingly targeted measures would make the practical recommendations concrete rather than boilerplate. Related to this, please clarify the applicability of V7 to the case building: under Chinese code, refuge floors are mandated only for buildings exceeding 100 m, whereas the case building is 49.15 m; explain how experts were instructed to score an indicator that is not normatively required for the assessed object.

7. There is an internal inconsistency in equation cross-referencing and a residual typesetting issue. In Section 2.2.3, the text states "Solving the linear system in Eq. (15)" when the linear system is actually Eq. (14). In addition, the specific game-theoretic combination coefficients (0.3571/0.6429 for the primary indices and 0.3122/0.6878 for the secondary indices) that you provided in the response letter should also appear in the manuscript or Supporting Information, since readers will not have access to the response document. Please also verify that all matrices in the SI are numbered consistently with the citations in the main text (Appendix Tables A1–A10 versus "Appendix Tables 1-5/1-10" as variously referenced).

8. The English has improved but still requires careful polishing. A few examples: "These dates point to the need..." (line 49) should read "These data point to..."; "Determination the standard cloud of assessment" should be "Determination of the standard cloud"; missing spaces after periods appear in several places (e.g., "related equipment.Meanwhile" around line 181); the descriptor "Fire risk is mediocre" in Table 5 is not idiomatic and should be "moderate"; and full-width punctuation and parentheses are mixed with half-width forms in Tables 1 and 2 and in several equations. The Conclusions also contain a sentence lacking terminal punctuation ("It reflects the specific characteristics of high-rise building fires"). A final round of professional language editing is recommended.

Reviewer #3: Literature & innovation: Briefly compare your index framework with the commercial complex assessment paper in Discussion, highlight unique high-rise vertical evacuation & external rescue indicators to further distinguish incremental contributions; trim repetitive descriptions of disaster risk theory in Introduction and Section 2.1.

Calculation transparency: Add inter-rater consistency metric of 12 experts in SI; repeat cloud similarity calculation 1–2 times in appendix to prove stability; unify mathematical symbol format of FAHP compatibility index and game theory weight equations, fix garbled formulas.

Case & comparison: In model comparison section, add simple statistical analysis of score deviation between your cloud model and FRI method; briefly explain the limitation of single residential case and clearer multi-scenario future research outlook.

Format & language: Full proofread English grammar and professional terms; revise inconsistent figure captions, table footnotes and missing unit labels; unify abbreviation definitions of all index dimensions at first occurrence.

Supplementary material: Sort FAHP, entropy weight raw tables orderly in Supporting Information, add clear table titles for all appendix calculation data for easy review.

After finishing above adjustments, the manuscript can meet PLOS ONE publication standards.

"Polish the language of the article to make it more concise and clear.

How can the content of the article be further streamlined?

Are there any specific examples to help the author better understand the minor revision suggestions?"

**********

what does this mean?). If published, this will include your full peer review and any attached files.

If you choose “no”, your identity will remain anonymous but your review may still be made public.

**Do you want your identity to be public for this peer review?**  For information about this choice, including consent withdrawal, please see our Privacy Policy

Reviewer #2: No

Reviewer #3: No

**********

[NOTE: If reviewer comments were submitted as an attachment file, they will be attached to this email and accessible via the submission site. Please log into your account, locate the manuscript record, and check for the action link "View Attachments". If this link does not appear, there are no attachment files.]

To ensure your figures meet our technical requirements, please review our figure guidelines: https://journals.plos.org/plosone/s/figures

You may also use PLOS’s free figure tool, NAAS, to help you prepare publication quality figures: https://journals.plos.org/plosone/s/figures#loc-tools-for-figure-preparation.

NAAS will assess whether your figures meet our technical requirements by comparing each figure against our figure specifications.

# Author comment: Author response to Decision Letter 2

**DOI:** 10.1371/journal.pone.0358941.r004

11 Aug 2026

Reviewer#2, Concern # 1: The stability verification of the cloud similarity calculation is still incomplete. You now report that 4,000 cloud drops were generated and that the Grade II similarity (0.5898) is far higher than the adjacent grades, which is reassuring. Nevertheless, the forward cloud generator is a stochastic procedure, and a single run does not demonstrate reproducibility. Please repeat the similarity calculation a reasonable number of times (e.g., 10–30 independent runs) and report the mean and standard deviation of the similarity values for each grade, or at minimum show that the grade assignment is invariant across repeated runs. This can be added as a short paragraph in Section 3.2.3 or in the Supporting Information.

Author response: Thank you for this valuable suggestion. We repeated the cloud similarity calculation independently 10 times while keeping the original assessment data, cloud parameters, and number of cloud drops (4,000 drops) unchanged. The mean values and standard deviations of the similarity values for each risk grade were calculated. The results show that the mean similarity values for Grades I–V were 0.0029, 0.5906, 0.0045, 0, and 0, respectively, with corresponding standard deviations of 0.0006, 0.0062, 0.0008, 0, and 0. The Grade II similarity remained consistently dominant in all repeated calculations, and the final risk grade assignment was unchanged as Grade II. These results demonstrate that the stochastic variation introduced by the forward cloud generator has a negligible influence on the final grade determination.

Author action: We have revised Section 3.2.3 of the main manuscript (Lines 475–478) and added the corresponding cloud similarity calculation results in the Supporting Information (Appendix Table A11).________________________________________

Reviewer#2, Concern # 2: The justification for the hyper-entropy setting He = 0.5 remains essentially an appeal to convention. Since He controls the "thickness" of the standard clouds and could in principle affect the similarity values near grade boundaries, I suggest adding a brief sensitivity analysis in which He is varied over a plausible range (e.g., 0.1–1.0) to show that the final grade determination is insensitive to this parameter. Given that your comprehensive cloud lies well inside the Grade II interval, I expect this analysis to be straightforward and to strengthen the robustness claim considerably.

Author response: Thank you for this valuable suggestion. The hyper-entropy values of the standard clouds were varied from 0.1 to 1.0 with an interval of 0.1, while all other parameters, including the comprehensive cloud characteristics and cloud generation settings, remained unchanged. The results showed that although the similarity values varied slightly under different hyper-entropy settings, Grade II consistently exhibited the highest similarity among all risk grades. Specifically, the similarity values of Grade II ranged from 0.5818 to 0.5979, which remained substantially higher than those of the adjacent grades. Therefore, the final fire risk grade remained unchanged as Grade II throughout the sensitivity analysis. These results demonstrate that the proposed assessment model is robust against reasonable variations in the hyper-entropy parameter, and the selection of He=0.5 does not artificially determine the final assessment result.

Author action: We have revised Section 3.2.3 of the main manuscript (Line 478) and added the results of the hyper-entropy sensitivity analysis in the Supporting Information (Appendix Table A12).________________________________________

Reviewer#2, Concern # 3: Although the composition and experience of the 12 experts are now described, the scoring protocol itself is still insufficiently documented. Please clarify: (a) whether the experts scored independently or after group discussion; (b) whether a written scoring rubric or anchoring descriptions (e.g., what conditions correspond to a score of 60 versus 80 for a given indicator) was provided; and (c) some quantitative measure of inter-rater agreement, such as Kendall's W or the coefficient of variation per indicator. Table 6 shows non-trivial dispersion for several indicators (e.g., E3 ranges from 69 to 96), so a consistency statistic would help readers judge the reliability of the expert input.

Author response: Thank you for this valuable comment. In this study, the 12 experts independently scored each indicator based on the actual conditions of the case building and their professional knowledge. Before scoring, the experts were provided with the definitions of all indicators and the corresponding risk-level intervals listed in Table 5. The independent scores were directly used as inputs for the cloud model to characterize the uncertainty and fuzziness of expert judgments. Regarding the inter-rater agreement analysis, we appreciate the reviewer’s suggestion. However, Kendall’s W or coefficient of variation was not adopted as a strict agreement measure in this study. The reason is that the expert panel consisted of specialists from different professional fields, and differences in their evaluations are expected because different experts may emphasize different aspects of high-rise building fire risk. These differences were considered meaningful information reflecting the diversity of expert knowledge rather than inconsistent judgments. The cloud model was specifically adopted to incorporate such randomness and fuzziness in expert evaluations.

Author action: We have revised Section 3.2 of the main manuscript (Lines 450-455).

________________________________________

Reviewer#2, Concern # 4: The quantification basis for the newly added exposure indicators E4 (tangible assets) and E5 (intangible assets) needs elaboration. These are conceptually sound additions, but it is unclear what information the experts relied on when scoring them for a residential building of this type—was any estimate of property value, business interruption potential, or replacement cost made available to the panel? A sentence or two describing the evidence base for these two indicators would prevent the impression that they were scored purely impressionistically.

Author response: Thank you for this valuable comment. For E4 (tangible assets), the experts considered the direct economic value associated with the building, including the estimated property value, construction investment cost, building scale, structural characteristics, and the potential direct losses of building components, equipment, facilities, and indoor property caused by fire. For E5 (intangible assets), considering that the case building is a residential high-rise building with commercial functions on the lower floors, the experts did not evaluate this indicator solely based on traditional business interruption losses. Instead, they considered the potential interruption of residential functions, impacts on ground-floor commercial services, social influence, recovery requirements, and possible changes in property value after similar fire events.

Author action: We have revised Section 2.1 of the main manuscript (Lines 205-207 , lines 209-211).

________________________________________

Reviewer#2, Concern # 5: The comparison with the FRI method, while expanded, still overstates what the exercise demonstrates. The linear normalization of the FRI score (3.579 on a 1–5 scale mapped to 71.58) should be stated explicitly in the text, including the direction of the scale (i.e., confirmation that higher FRI values correspond to lower risk, so that the two scales are commensurable). Moreover, agreement between two expert-judgment-based methods applied by the same investigators to the same building constitutes consistency, not independent validation. I recommend softening the wording ("confirming the validity") in the Abstract and Conclusions to something like "supporting the plausibility/consistency of the proposed model," and acknowledging that both methods share partially overlapping subjective inputs.

Author response: Thank you for this valuable comment. The linear normalization procedure of the FRI score was already described in the original manuscript (Lines 490–497), corresponding to Lines 547–549 in the revised manuscript. In the revised manuscript, we further clarified the meaning and direction of the FRI score, indicating that higher FRI scores correspond to better fire safety performance and lower fire risk levels. The FRI risk classification criteria were also added. In addition, the descriptions in the Abstract, Methods, and Model Comparison sections were revised to avoid overstating the validation capability of the proposed model. The comparison with the FRI method is now interpreted as supporting the plausibility and consistency of the proposed assessment framework.

Author action: We revised the relevant descriptions in the Abstract (Lines 28–29), Methods (Line 149-150), Model Comparison section (Lines 553–557), and Conclusions (Lines 577–578).

________________________________________

Reviewer#2, Concern # 6: Section 3.3 remains rather generic. The value of an indicator-based model lies precisely in its diagnostic capability, yet the analysis discusses only dimension-level weights. Please link the discussion to the actual case results: for example, V7 (refuge floors and facilities, Ex = 67.5), V1 (proportion of elderly and children, Ex = 69.25), and E1 (population size, Ex = 69.83) received the lowest expert scores, which is notable given that more than half of the residents are reported to be elderly or under 14. Identifying these weak items and proposing correspondingly targeted measures would make the practical recommendations concrete rather than boilerplate. Related to this, please clarify the applicability of V7 to the case building: under Chinese code, refuge floors are mandated only for buildings exceeding 100 m, whereas the case building is 49.15 m; explain how experts were instructed to score an indicator that is not normatively required for the assessed object.

Author response: Thank you for your valuable suggestion. In the revised manuscript, we have supplemented the indicator-level analysis based on the actual assessment results of the case-study building. Specifically, the indicators with relatively low expert evaluation values, including V7, V1, and E1, were identified and further discussed. The revised discussion explains the specific risk implications of these weak indicators and proposes targeted improvement measures. Regarding V7, we appreciate the reviewer’s comment on its applicability. The purpose of this indicator is not to evaluate only the mandatory installation of refuge floors, but to assess the overall refuge-related evacuation support capacity of high-rise buildings. For buildings below 100 m, such as the case-study building (49.15 m), experts were instructed to evaluate V7 mainly based on the availability, accessibility, and effectiveness of applicable refuge-related and auxiliary evacuation facilities. For buildings exceeding 100 m, both refuge floors and related refuge facilities should be considered. Therefore, the V7 indicator can be applied to different types of high-rise buildings by considering their specific structural characteristics and evacuation requirements. (the configuration and requirements of such escape apparatus are specified in GB 21976, Escape Apparatus for Building Fire).

Author action: We have revised Section 3.3 of the manuscript (lines 493–511).

________________________________________

Reviewer#2, Concern # 7: There is an internal inconsistency in equation cross-referencing and a residual typesetting issue. In Section 2.2.3, the text states "Solving the linear system in Eq. (15)" when the linear system is actually Eq. (14). In addition, the specific game-theoretic combination coefficients (0.3571/0.6429 for the primary indices and 0.3122/0.6878 for the secondary indices) that you provided in the response letter should also appear in the manuscript or Supporting Information, since readers will not have access to the response document. Please also verify that all matrices in the SI are numbered consistently with the citations in the main text (Appendix Tables A1–A10 versus "Appendix Tables 1-5/1-10" as variously referenced).

Author response: Thank you for this valuable comment. The incorrect cross-reference in Section 2.2.3 has been corrected, and the equation numbering has been carefully checked throughout the manuscript. Regarding the game-theoretic combination coefficients, the calculation procedure, relevant equations, and the input weight vectors obtained from FAHP and SEWM have already been provided in the manuscript and Supporting Information. Therefore, the combination coefficients can be directly derived according to the described method. In addition, the Supporting Information has been carefully checked, and the numbering of supplementary tables has been unified as Appendix Tables A1–A12 to ensure consistency with the citations in the main text.

Author action: We have corrected errors in Section 2.2.3 (line 352) and the Supporting Information.________________________________________

Reviewer#2, Concern # 8: The English has improved but still requires careful polishing. A few examples: "These dates point to the need..." (line 49) should read "These data point to..."; "Determination the standard cloud of assessment" should be "Determination of the standard cloud"; missing spaces after periods appear in several places (e.g., "related equipment. Meanwhile" around line 181); the descriptor "Fire risk is mediocre" in Table 5 is not idiomatic and should be "moderate"; and full-width punctuation and parentheses are mixed with half-width forms in Tables 1 and 2 and in several equations. The Conclusions also contain a sentence lacking terminal punctuation ("It reflects the specific characteristics of high-rise building fires"). A final round of professional language editing is recommended.

Author response: Thank you for your valuable suggestion. We have carefully revised the manuscript and conducted a comprehensive language and formatting check. In addition, full-width and half-width punctuation marks have been standardized throughout the manuscript, and the overall English expression has been further polished.

Author action: The manuscript has been thoroughly proofread and revised accordingly.

________________________________________

Reviewer#3, Concern # 1: Literature & innovation: Briefly compare your index framework with the commercial complex assessment paper in Discussion, highlight unique high-rise vertical evacuation & external rescue indicators to further distinguish incremental contributions; trim repetitive descriptions of disaster risk theory in Introduction and Section 2.1.

Author response: Thank you for your valuable suggestion. In the revised manuscript, we further clarified the differences between the proposed index framework and previous fire risk assessment frameworks for large commercial buildings and shopping malls. Compared with previous studies, the proposed framework places greater emphasis on the specific evacuation and rescue characteristics of high-rise buildings, particularly vertical evacuation-related indicators and city-level external rescue capacity. These considerations better reflect the long vertical evacuation distances and greater dependence on professional rescue forces in high-rise building fires, thereby further distinguishing the incremental contribution of the proposed framework. In addition, we streamlined the descriptions of Integrated Disaster Risk Theory in the Introduction and Section 2.1 to reduce repetition.

Author action: We streamlined the description of Integrated Disaster Risk Theory in Section 2.1 (lines 158–163) and added a brief comparison with previous fire risk assessment frameworks for large commercial buildings and shopping malls in Section 3.3, “Analysis of the assessment results” (lines 512–517). ________________________________________

Reviewer#3, Concern # 2: Calculation transparency: Add inter-rater consistency metric of 12 experts in SI; repeat cloud similarity calculation 1–2 times in appendix to prove stability; unify mathematical symbol format of FAHP compatibility index and game theory weight equations, fix garbled formulas.

Author response: Thank you for your valuable suggestion. The 12 experts independently evaluated each indicator based on the actual conditions of the case building, the indicator definitions, and the corresponding risk-level intervals. Considering the different professional backgrounds of the experts, differences in their evaluations were regarded as meaningful information reflecting the diversity of expert knowledge rather than being treated solely as inconsistency. Therefore, Kendall’s W or the coefficient of variation was not adopted as a strict inter-rater agreement measure, while the randomness and fuzziness of the expert assessments were incorporated through the cloud model. In addition, the cloud similarity calculation was independently repeated 10 times under unchanged assessment data, cloud parameters, and number of cloud drops. The results showed that Grade II consistently had the highest similarity in all repeated calculations, with a mean similarity of 0.5906 and a standard deviation of 0.0062, confirming the stability of the final grade determination. The mathematical symbols and equation formats for the FAHP compatibility index and game-theoretic combination weighting were also carefully checked and standardized, and the garbled or inconsistent formulas were corrected.

Author action: We added the results of 10 independent repeated cloud similarity calculations in the Supporting Information (Appendix Table A11) and standardized the mathematical symbols and equation formats for the FAHP compatibility index and game-theoretic combination weighting.________________________________________

Reviewer#3, Concern # 3: Case & comparison: In model comparison section, add simple statistical analysis of score deviation between your cloud model and FRI method; briefly explain the limitation of single residential case and clearer multi-scenario future research outlook.

Author response: Thank you for your valuable suggestion. In the Model Comparison section, we further quantified the deviation between the two assessment results. After normalization of the FRI score to a 0–100 scale, the FRI result was 71.58, compared with the comprehensive-cloud expectation value of 78.6933. The absolute deviation was 7.1133, corresponding to a relative deviation of approximately 9.04%. Although a numerical difference exists, both methods yielded the same qualitative conclusion of relatively low fire risk. In addition, we clarified the limitation associated with using a single high-rise residential building and further emphasized the need for validation using buildings with different functions, heights, regions, and management conditions in future research.

Author action: The comparison analysis has been supplemented with the statistical deviation analysis between the proposed cloud model and the FRI method. The limitations of the single case study and the future research prospects for multi-scenario applications have also been added to the revised manuscript. ________________________________________

Reviewer#3, Concern # 4: Format & language: Full proofread English grammar and professional terms; revise inconsistent figure captions, table footnotes and missing unit labels; unify abbreviation definitions of all index dimensions at first occurrence.

Author response: Thank you for your valuable suggestion. We have carefully proofread the revised manuscript to improve English grammar, academic expressions, and the consistency of professional terminology. In addition, we have checked and revised the figure captions, table descriptions, and unit labels throughout the manuscript where necessary.

Author action: The manuscript has been carefully proofread and revised for grammar, professional terminology, figure captions, table descriptions, and unit labels. ________________________________________

Reviewer#3, Concern # 5: Supplementary material: Sort FAHP, entropy weight raw tables orderly in Supporting Information, add clear table titles for all appendix calculation data for easy review.

Author response: Thank you for your valuable suggestion. The FAHP-related calculation tables (Appendix Tables A1–A5) and SEWM-related calculation tables (Appendix Tables A6–A10) have been arranged in a sequential order according to the indicator hierarchy. In addition, the titles of all appendix tables have been revised to improve clarity and allow readers to identify the corresponding calculation data more easily.

Author action: We reorganized the supplementary calculation tables and revised the titles of Appendix Tables A1–A12 in the Supporting Information. The FAHP, SEWM, cloud similarity calculation, and sensitivity analysis data are now presented in a clearer and more systematic order.

Attachment Submitted filename: Response_to_Reviewers_auresp_2.docx

# Peer review: Decision Letter 2

**DOI:** 10.1371/journal.pone.0358941.r005

**Authors:** Yang (Jack) Lu

8 Sep 2026

Fire risk assessment for high-rise buildings based on Integrated Disaster Risk Theory

PONE-D-26-13643R2

Dear Dr. liu,

We’re pleased to inform you that your manuscript has been judged scientifically suitable for publication and will be formally accepted for publication once it meets all outstanding technical requirements.

Within one week, you’ll receive an e-mail detailing the required amendments. When these have been addressed, you’ll receive a formal acceptance letter and your manuscript will be scheduled for publication.

An invoice will be generated when your article is formally accepted. Please note, if your institution has a publishing partnership with PLOS and your article meets the relevant criteria, all or part of your publication costs will be covered. Please make sure your user information is up-to-date by logging into Editorial Manager at Editorial Manager® and clicking the ‘Update My Information' link at the top of the page. For questions related to billing, please contact billing support.

If your institution or institutions have a press office, please notify them about your upcoming paper to help maximize its impact. If they’ll be preparing press materials, please inform our press team as soon as possible -- no later than 48 hours after receiving the formal acceptance. Your manuscript will remain under strict press embargo until 2 pm Eastern Time on the date of publication. For more information, please contact onepress@plos.org.

Kind regards,

Yang (Jack) Lu, PhD

Academic Editor

PLOS One

Additional Editor Comments (optional):

Reviewers' comments:

Reviewer's Responses to Questions

**Comments to the Author**

Reviewer #2: All comments have been addressed

Reviewer #4: All comments have been addressed

**********

2. Is the manuscript technically sound, and do the data support the conclusions?

Reviewer #2: (No Response)

Reviewer #4: Yes

**********

3. Has the statistical analysis been performed appropriately and rigorously?

Reviewer #2: (No Response)

Reviewer #4: Yes

**********

4. Have the authors made all data underlying the findings in their manuscript fully available?

The PLOS Data policy

Reviewer #2: (No Response)

Reviewer #4: Yes

**********

5. Is the manuscript presented in an intelligible fashion and written in standard English?

Reviewer #2: (No Response)

Reviewer #4: Yes

**********

Reviewer #2: I am happy to suggest accepting this manuscript without revisions. The research design is rigorous, analytical work is robust, and all results are well organized and clearly illustrated. This paper offers significant contributions to the field, and I have no further comments to raise.

Reviewer #4: The manuscript entitled “Fire risk assessment for high-rise buildings based on Integrated Disaster Risk Theory” presents a comprehensive and systematic framework for evaluating fire risks in high-rise buildings by integrating Integrated Disaster Risk Theory, game-theoretic combination weighting, and cloud model theory. The topic addressed in this study is highly relevant because high-rise buildings involve complex fire safety challenges, including difficulties in fire suppression, diverse occupancy characteristics, rapid fire propagation, and evacuation limitations, which may result in severe casualties and property losses if effective risk assessment and prevention strategies are lacking. The manuscript successfully responds to this practical challenge by developing a more systematic and scenario-specific fire risk assessment approach.

The major strength of this study is the construction of a high-rise building-specific fire risk assessment indicator system based on Integrated Disaster Risk Theory. Unlike traditional assessment methods that mainly emphasize fire hazards, building vulnerability, or fire protection facilities, the proposed framework considers the complete risk formation process through four dimensions: hazard, exposure, vulnerability, and emergency response and recovery capacity. The framework further incorporates high-rise-specific factors, such as vertical evacuation difficulties, evacuation facilities, asset exposure, and external rescue capacity, thereby improving the comprehensiveness and applicability of the assessment model.

The methodological framework is scientifically sound. The authors combine FAHP and the structure entropy weight method through a game-theoretic combination weighting strategy, which effectively integrates subjective expert judgment and objective weighting information while reducing potential bias from a single weighting method. In addition, the cloud model is appropriately introduced to address uncertainty and fuzziness in expert evaluation, enabling the transformation of qualitative risk judgments into quantitative assessment results. The proposed methodology therefore provides a reasonable and robust solution for complex fire risk assessment problems.

The empirical analysis and validation procedures provide sufficient support for the conclusions. The authors apply the proposed framework to a typical high-rise residential building in Changchun and obtain a comprehensive cloud result with Ex = 78.6933, En = 4.4848, and He = 1.2983. Based on repeated cloud similarity calculations, the building is consistently classified as Grade II fire risk, indicating a relatively low overall fire risk level. The revised manuscript further strengthens the reliability of the model by conducting ten independent repeated cloud similarity calculations, demonstrating that Grade II remains the dominant classification with stable similarity values, confirming that stochastic variation does not significantly influence the final assessment result. Moreover, the hyper-entropy sensitivity analysis confirms that reasonable variations in model parameters do not change the final risk classification, supporting the robustness of the proposed framework.

The comparison with the established Fire Risk Index (FRI) method also supports the feasibility and consistency of the proposed approach. Both methods reached the same qualitative conclusion regarding the relatively low fire risk of the case-study building. Importantly, the authors appropriately revised the interpretation of this comparison and clarified that it demonstrates consistency and plausibility rather than independent validation, which improves the scientific rigor of the manuscript.

The revised manuscript has adequately addressed the major concerns raised during peer review. The authors improved the transparency of expert scoring procedures, clarified the basis for newly introduced exposure indicators, enhanced the discussion of indicator-level risk factors, corrected mathematical and formatting issues, and improved the overall language quality. The additional analysis of weak indicators, including V7, V1, and E1, provides practical value by identifying specific risk-control priorities rather than only reporting an overall risk grade. Furthermore, the authors provide a balanced discussion of limitations, acknowledging that the current validation is based on a single high-rise residential building and that future studies should extend validation to buildings with different functions, heights, regions, and management conditions.

Overall, this manuscript presents a valuable contribution to high-rise building fire risk assessment by combining a solid theoretical foundation, an appropriate uncertainty-handling methodology, and a practical case application. The proposed framework demonstrates methodological innovation, analytical reliability, and practical significance for fire prevention, evacuation management, and emergency rescue planning. The revisions have substantially improved the quality and clarity of the manuscript, and no major issues remain that would prevent publication.

**********

what does this mean?). If published, this will include your full peer review and any attached files.

If you choose “no”, your identity will remain anonymous but your review may still be made public.

**Do you want your identity to be public for this peer review?**  For information about this choice, including consent withdrawal, please see our Privacy Policy

Reviewer #2: No

Reviewer #4: No

**********

# Editor report: Acceptance letter

**DOI:** 10.1371/journal.pone.0358941.r006

**Authors:** Yang (Jack) Lu

PONE-D-26-13643R2

PLOS One

Dear Dr. Liu,

I'm pleased to inform you that your manuscript has been deemed suitable for publication in PLOS One. Congratulations! Your manuscript is now being handed over to our production team.

At this stage, our production department will prepare your paper for publication. This includes ensuring the following:

* All references, tables, and figures are properly cited

* All relevant supporting information is included in the manuscript submission,

* There are no issues that prevent the paper from being properly typeset

You will receive further instructions from the production team, including instructions on how to review your proof when it is ready. Please keep in mind that we are working through a large volume of accepted articles, so please give us a few days to review your paper and let you know the next and final steps.

Lastly, if your institution or institutions have a press office, please let them know about your upcoming paper now to help maximize its impact. If they'll be preparing press materials, please inform our press team within the next 48 hours. Your manuscript will remain under strict press embargo until 2 pm Eastern Time on the date of publication. For more information, please contact onepress@plos.org.

You will receive an invoice from PLOS for your publication fee after your manuscript has reached the completed accept phase. If you receive an email requesting payment before acceptance or for any other service, this may be a phishing scheme. Learn how to identify phishing emails and protect your accounts at https://explore.plos.org/phishing.

If we can help with anything else, please email us at customercare@plos.org.

Thank you for submitting your work to PLOS One and supporting open access.

Kind regards,

PLOS One Editorial Office Staff

on behalf of

Dr. Yang (Jack) Lu

Academic Editor

PLOS One
